# Anxiolytic-Like Effect of *Hyptis crenata* Essential Oil: Behavioral Insights and *In Silico* SERT Modulation

**DOI:** 10.1021/acsomega.5c11201

**Published:** 2026-03-06

**Authors:** Savyo Mikael Lacerda Gomes, André Nogueira Cardeal dos Santos, José Lucas Gomes Izidorio, José Ednésio da Cruz Freire, Kirley Marques Canuto, Marília Cavalcante Araújo, Francisco Sydney Henrique Félix, Marcus Vinícius Vieira Torquato, Jonathan Elias Rodrigues Martins, Yuri Abreu Gomes-Vasconcelos, Kerly Shamyra Silva Alves, José Henrique Leal Cardoso, José Eduardo Ribeiro Honório Júnior, Andrelina Noronha Coelho de Souza

**Affiliations:** † Experimental Physiology Laboratory, State University of Ceara, Superior Institute of Biomedical Sciences, Ave. Dr. Silas Munguba -1700, 60714-903 Fortaleza, Ceara, Brazil; ‡ Neuroscience and Translational Medicine Laboratory, Christus University - Unichristus, St. João Adolfo Gurgel − 133, 60192-345 Fortaleza, Ceara, Brazil; § Biochemistry and Gene Expression Laboratory, State University of Ceara, Superior Institute of Biomedical Sciences, Ave. Dr. Silas Munguba -1700, 60714-903 Fortaleza, Ceara, Brazil; ∥ Laboratory of Natural Products Chemistry, Embrapa Tropical Agroindustry, Brazilian Agricultural Research Corporation, St. Doutora Sara Mesquita − 2270, 60511-110 Fortaleza, Ceara, Brazil; ⊥ Electrophysiology Laboratory, State University of Ceará, Superior Institute of Biomedical Sciences, Ave. Dr. Silas Munguba -1700, 60714-903 Fortaleza, Ceara, Brazil

## Abstract

**Purpose:** The essential oil of *Hyptis crenata* (EOHc) contains several terpenes, many of which have anxiolytic-like activity. Thus, the aim of this study was to extract, analyze the chemical composition, and evaluate the anxiolytic-like effect of EOHc in mice using experimental approaches (behavioral parameters) and *in silico* studies. **Methods:** Mice were divided into seven experimental groups: Group I (no stress); Group II, stress only (no treatment); Group III, Tween 80 (stress and 0.1%, vehicle); Group IV, Tween 80 (no stress and 0.1%, vehicle); Group V, mirtazapine (stress and 30 mg/kg); Group VI, citalopram (stress and 10 mg/kg); Group VII, essential oil of *H. crenata* (stress and EOHc, 100 mg/kg); Group VIII, essential oil of *H. crenata* (no stress and EOHc, 100 mg/kg); Group IX, essential oil of *H. crenata* (stress and EOHc, 300 mg/kg); and Group X, essential oil of *H. crenata* (no stress and EOHc, 300 mg/kg);. After eight stress days, the behavioral tests (open field, elevated plus maze, and RotaRod) were started. Furthermore, docking and molecular dynamics analysis were used. To evaluate the safe administration of EOHc, water consumption, food consumption, and weight parameters were evaluated during 7 days of treatment. **Results**: The oil yield was 1.0–1.5%. Chromatography revealed that the top 5 constituents were caryophyllene V1, azulene, α-pinene, bornanone, and viridiflorene. All the treatments reduced the crossover and the rearing (*p*-value ≤ 0.05; ANOVA; Tukey’s post hoc test). During grooming time, neither stress nor treatments induced any changes. The stress altered the number of entries and the time spent in both open arm and closed effect that was reversed by all treatments except mirtazapine (*p* ≤ 0.05; ANOVA; Tukey’s test). There was no significance in the evaluation of water consumption, food consumption, and weight. With the exception of the Tween group, the RotaRod Test showed no significance between the control, 100 mg/kg EOHc, and 300 mg/kg EOHc groups. **Conclusion:** It can be concluded that EOHc presents an anxiolytic effect experimentally, with the probable inhibition of Apo SERT, due to the terpenoid constituents present in the oil.

## Graphical abstract

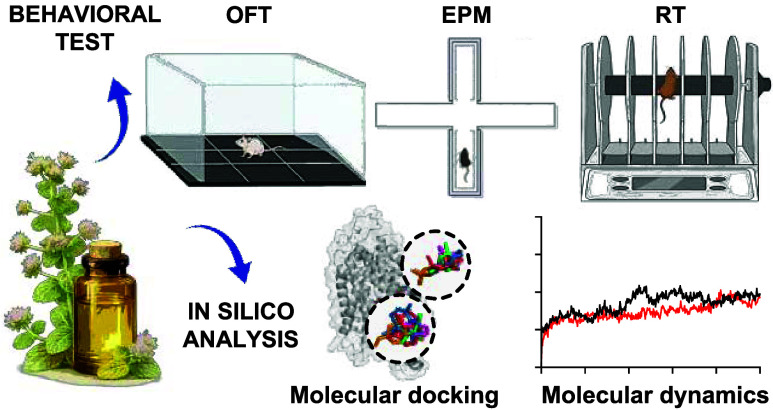

## Introduction

Anxiety disorders (ADs) represent a major global mental health concern, characterized by high prevalence and significant psychosocial burden.^1–4^ Individuals affected by ADs often experience reduced occupational performance and impairments in familial and social functioning, frequently necessitating clinical intervention.^5,6^ Epidemiological evidence indicates that Brazil ranks among the countries with the highest reported prevalence of AD, affecting approximately 18 million individuals.^7^ Notably, a 25.6% increase in reported cases has been attributed to the COVID-19 pandemic, with a higher incidence observed in women.^8^ The clinical manifestations of AD encompass persistent fear, apprehension, and heightened nervousness, often disproportionate to external stimuli.^9^ Moreover, the chronicity of these symptoms serves as a key diagnostic parameter distinguishing pathological anxiety from transient anxious states.^10^

The heterogeneity of AD symptoms presents a significant therapeutic challenge, frequently resulting in chronic or recurrent episodes. Current pharmacological interventions include selective serotonin reuptake inhibitors (SSRIs), serotonin–norepinephrine reuptake inhibitors (SNRIs), benzodiazepines, and β-adrenergic antagonists.^11–14^ Despite their clinical efficacy, these drugs are associated with a wide spectrum of adverse effects, including cognitive impairment, sedation, gastrointestinal disturbances, hepatic dysfunction, leukopenia, tolerance, and dependence.^15,16^ Consequently, the development of novel anxiolytic agents with improved safety profiles and faster onset of action remains an urgent pharmacological priority.

In recent years, natural products, particularly plant-derived essential oils, have garnered increasing attention as potential anxiolytic candidates due to their favorable toxicological profiles, affordability, and multifactorial mechanisms of action.^17^ *Hyptis crenata* Pohl ex Benth. (Lamiaceae), commonly known as “salva-do-marajó,” “hortelã-do-campo,” or “Brazilian mint,” is an aromatic species traditionally used in Brazilian folk medicine.^18^ Its essential oil exhibits a broad spectrum of biological activities, including anti-inflammatory, gastroprotective, and hepatoprotective effects.^19,20^ Given the pharmacological potential of the *H. crenata* essential oil (EOHc), the present study aimed to (i) extract and characterize its chemical constituents and (ii) evaluate its putative anxiolytic effects through both *in vivo* behavioral assays in *Mus musculus* (Swiss strain) subjected to chronic stress and complementary *in silico* modeling approaches.

## Materials and Methods

### Animals

Male *M. musculus* (Swiss strain) were used in the experimental protocol. A total of 100 four-week-old mice were randomly assigned into 10 groups containing 10 animals each. The mice were housed in polypropylene cages under controlled environmental conditions (26 ± 2 °C; 12-h light/dark cycle) and provided with standard rodent chow (Purina) and water *ad libitum*. All experimental procedures complied with the Guide for the Care and Use of Laboratory Animals and the ethical standards of the Brazilian Society of Laboratory Animal Science (SBCAL). Ethical approval was obtained from the Animal Research Ethics Committee of Unichristus University (protocol No. 027/23).

Group I served as the negative control (no stress and no treatment). The remaining groups were subjected to a chronic stress protocol lasting 14 days, after which oral treatments were administered by gavage for seven consecutive days starting on day 8. The experimental design comprised the following conditions: Group II received stress only without treatment; Group III was exposed to stress and received the vehicle (Tween 80, 0.1%); Group IV received the vehicle alone without stress exposure; Group V was treated with mirtazapine (30 mg/kg) under stress; Group VI received citalopram (10 mg/kg) under stress; Groups VII and IX received the EOHc under stress at doses of 100 and 300 mg/kg, respectively; and Groups VIII and X received the same doses of EOHc (100 and 300 mg/kg) without stress exposure. All solutions, including the essential oil formulations, were prepared using Tween 80 (0.1%) as the vehicle. At the end of the experimental period, all animals were humanely euthanized by anesthetic overdose in accordance with the approved ethical guidelines.

### Extraction and EOHc Characterization

Aerial parts of *H. crenata* (dried leaves and branches) were collected in São Raimundo das Mangabeiras, Maranhão, Brazil, and dried at room temperature under light-protected conditions. The botanical identification had been previously validated, and the specimen was deposited under voucher number MFS006776 at the Marlene Freitas da Silva Herbarium (https://herbariomfs.uepa.br/colecao-biocultural/salva-do-marajo-exsicata/). The EOHc was obtained by steam-distillation using a Clevenger-type apparatus, with an extraction time of 1 h and 30 min for each 100 g of plant material. The oil was stored at 4 °C in amber glass bottles until further analysis, following previously established recommendations.^21^

The chemical composition of EOHc was analyzed at the Multi-User Natural Products Chemistry Laboratory (LMQPN) of Embrapa Tropical Agroindustry, Fortaleza, Ceará, Brazil. Gas chromatography–mass spectrometry (GC–MS) was performed using an Agilent 7890B gas chromatograph coupled to a 5977A mass selective detector (quadrupole) equipped with a flame ionization detector. Separation was achieved using an HP-5 MS methylpolysiloxane column (30 m × 0.25 mm × 0.25 μm; Agilent Technologies, Santa Clara, CA, USA). The injector temperature was set to 250 °C, the detector to 150 °C, and the transfer line to 280 °C. The oven temperature program started at 70 °C, increased at 4 °C/min up to 180 °C, and subsequently at 10 °C/min up to 250 °C, yielding a total run time of 34.5 min. Mass spectra were recorded in the 40–600 *m*/*z* range using MassHunter B.06.00 software (Agilent Technologies). Compound identification was performed by comparing the obtained spectra with reference data from the National Institute of Standards and Technology (NIST) library.

### Treatment of Animals with EOHc

For pharmacological treatment, aliquots of the EOHc were freshly prepared each day by dilution in saline solution containing 0.1% Tween 80 and administered orally by gavage at doses of 100 and 300 mg/kg. These concentrations were selected based on previous studies conducted by the research group, which demonstrated their safety and biological efficacy.^22^ The reference drugs mirtazapine and citalopram (Sanofi Medley, Campinas, São Paulo, Brazil) were also diluted in saline solution and administered at doses of 30 mg/kg and 10 mg/kg, respectively. Tween 80 used for vehicle preparation was obtained from Sigma-Aldrich (Saint Louis, Missouri, USA).

### Animal Induction to Chronic Stress

During a 15-day observation period, male *M. musculus* (Swiss strain) were subjected to a chronic unpredictable stress (CUS) paradigm consisting of alternating environmental and physical stressors. The stressors included a cage tilted at a 45° angle for 18 h, exposure to a wet cage for 12 h, food and water deprivation for 12 h, physical restraint for 1 h, inversion of the light/dark cycle, and continuous illumination. These stressors were applied in a randomized and unpredictable sequence to prevent habituation, following the chronic stress progression protocol originally described by Katz and Hersh and Katz et al.,^23,24^ with minor adaptations approved by the Unichristus Ethics Committee.

### Weight Monitoring, Water and Feed Consumption

Throughout the 7-day treatment period, animals were monitored daily for water intake, food consumption, and body weight variation. For the assessment of water intake, each cage received 50 mL of fresh water daily, and the volume consumed was determined by subtracting the residual amount measured 24 h later. Food consumption was evaluated by providing 200 g of standard chow per cage every 24 h, with the remaining food weighed to determine daily intake. Body weight was recorded once daily during the 7-day period, and mean values were calculated to evaluate possible variations associated with treatment or stress exposure.

### Behavioral Tests

To assess the behavioral effects of EOHc, animals were subjected to three validated paradigms: the Open Field Test (OFT), the Elevated Plus Maze (EPM), and the RotaRod test (RT). All tests were performed under controlled environmental conditions and conducted 60 min after the final administration of treatments (EOHc 100 or 300 mg/kg, citalopram 10 mg/kg, mirtazapine 30 mg/kg, or saline 10 mL/kg), ensuring consistent systemic exposure across groups.

The OFT was employed to evaluate locomotor and exploratory activity, following the protocol described by Walsh and Cummins (1976).^25^ Each mouse was individually placed in an acrylic arena (30 × 30 × 15 cm) with transparent walls and a black floor divided into nine equal quadrants. Animals were placed at the center of the arena and observed for 5 min following a 1 min habituation period to allow recognition of the test environment. The number of crossings, rearing events, and grooming behaviors were recorded as indicators of locomotor and exploratory activity. Between trials, the arena was thoroughly cleaned with 70% ethanol to eliminate olfactory cues and ensure experimental consistency.^26^

The EPM test was performed according to the methodology of Handley and Mithani (1984) to assess anxiety-like behavior. The apparatus consisted of two opposite open arms (30 cm × 5 cm × 25 cm) and two closed arms (30 cm × 5 cm × 25 cm) arranged perpendicularly around a central platform (5 cm × 5 cm). The maze, constructed of transparent acrylic with a black floor, was elevated 50 cm above the ground and placed in a dimly lit room. Each mouse was individually positioned at the center of the maze facing one of the closed arms and observed for 5 min. Behavioral parameters included the number of entries into the open and closed arms and the time spent in each, serving as indices of anxiety and exploratory drive.

Motor coordination and balance were evaluated using the RT, performed on a commercial device (Insight, model EFF-411) equipped with a nonslip rotating drum and fixed-speed control (Figure 1). Following a 60 min acclimatization period to the experimental room, the training and testing procedures were conducted over three consecutive days. On the first day, each animal underwent three trials at a rotation speed of 15 rpm, with a 300-s cutoff per trial and 15 min rest intervals. On the second day, the same protocol was repeated at 20 rpm, and on the third day, animals were tested at 25 rpm using identical trial conditions. This progressive-speed protocol, consisting of a gradual motor challenge, aligns with validated methodologies for assessing motor performance, coordination, and motor learning in rodents.^27,28^

**Figure 1:**
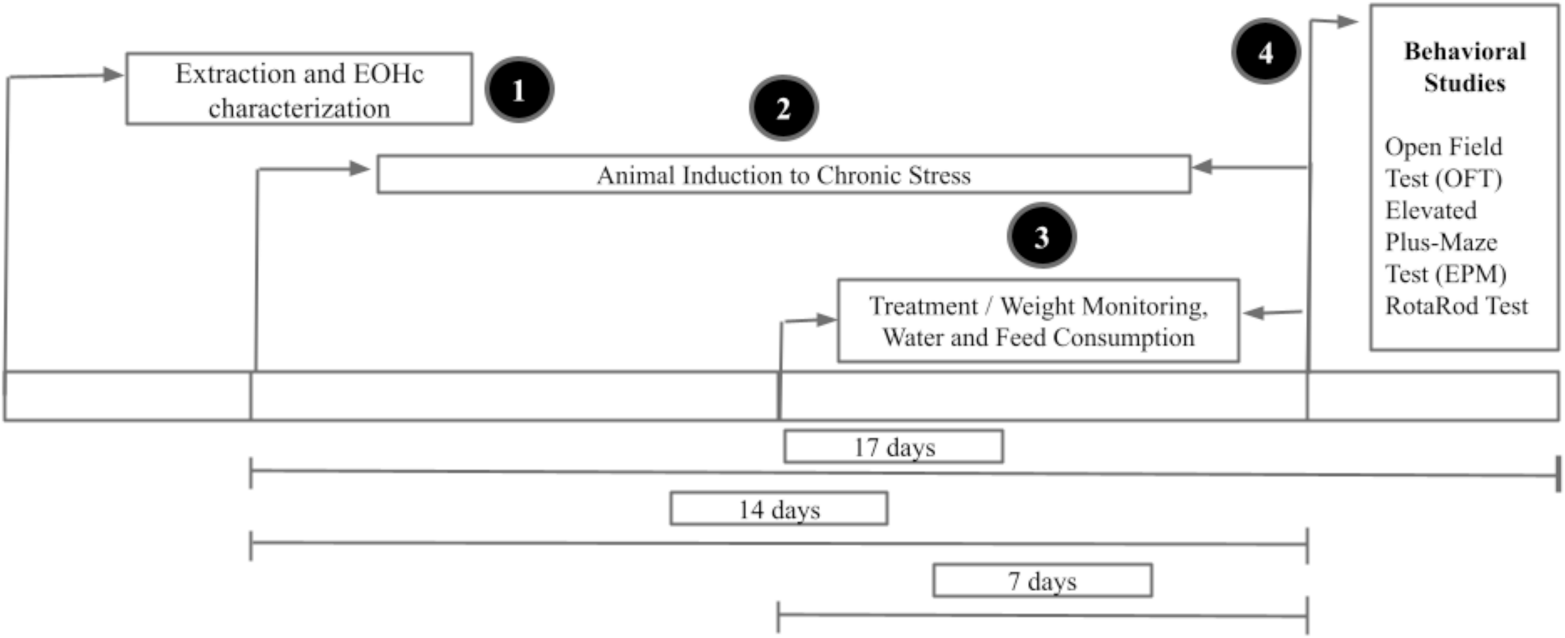
Experimental design of activities. 1: Beginning of the EOHc extraction and characterization process (first day); 2: Induction of chronic stress in mice (14 days of stress induction); 3: Beginning of treatments and monitoring of body weight, water, and food intake (7 days of treatment); 4: Experimental tests (OFT, EPM, and RT).

### Statistical Analysis

Statistical analysis was performed using the Google Colaboratory environment (Google Colab), with scripts developed in Python (version 3.10). The pandas, numpy, scipy.stats, and statsmodels libraries were used to process the data and perform the statistical tests. The databases were attached to a spreadsheet containing behavioral measurements collected experimentally in the OFT, EPM, and RT. The values obtained were organized into numerical lists corresponding to each experimental group. From these data, the means and standard errors of the mean were calculated. To assess statistical differences between groups, a one-way ANOVA (one-way ANOVA) was applied, followed by Tukey’s multiple comparisons test (HSD), implemented by the function. The significance level adopted was *p* < 0.05. Significant differences are indicated in the corresponding figures and tables.

### *In Silico* Insights into the Main Components of EOHc and apo SERT

#### Bioactivity Prediction Main Components of EOHc

The prediction of pharmacokinetic properties, drug-likeness, and potential toxicity of the five major constituents of EOHc (monoterpenes: α-pinene and bornanone; sesquiterpenes: caryophyllene V1, viridiflorene, and azulene) was performed using the ADMETlab 2.0,^29^ CODD-Pred,^30^ pKCSM,^31^ and SwissADME^32^ platforms, utilizing the SMILES format. All bioactivity analyses were conducted based on the criteria established by Lipinski’s and Veber’s rules, which suggest that these constituents should demonstrate the ability to cross the blood-brain barrier, exhibit permeability in the central nervous system, and show no neurotoxic, hepatotoxic, or nephrotoxic potential. Additionally, they should be nonmutagenic, noncarcinogenic, and should not inhibit or act as substrates for the hERG potassium ion channel, which is primarily involved in cardiac repolarization, as well as for enzymes belonging to the cytochrome P_450_ protein family (CYP_2D6_ and CYP_3A4_) and organic cation transporter 2 (OCT_2_) enzyme.^33^

### Molecular Docking and Molecular Dynamics Insight

Docking simulations were conducted using AutoDock Tools 1.5.6 and AutoDock Vina v. 1.1.2,^34^ utilizing the cocrystal structure of the serotonin transporter (5-HT transporter, apo SERT) complexed with *S*-citalopram and Br-citalopram (PDB ID: 5I75). To validate the molecular docking protocol, redocking simulations were initially performed, allowing all torsional bonds of the ligand and amino acids in the receptor’s catalytic site to rotate freely, as detailed in previous publications.^35,36^

In preparation, polar hydrogen atoms were added to the receptor and parametrized using Gasteiger charges. Following this validation, the docking predictions were configured as follows: polar hydrogen atoms were incorporated into the 5-HT transporter structure, and partial atomic charges were assigned using the Gasteiger method. Ligands: α-pinene (CID: 12223113), d-2-bornanone (CID: 9543187), caryophyllene V1 (CID: 564746), azulene (CID: 6432243), and viridiflorene (CID: 10910653) were parametrized with Gasteiger charges added. All torsional bonds of the ligands were permitted to rotate freely, while the 5-HT transporter remained rigid, except for the following residues: Tyr^95^, Trp^103^, Arg^104^, Ile^168^, Ala^169^, Ile^172^, Tyr^175^, Tyr^176^, Phe^334^, Phe^335^, Ser^336^, Leu^337^, Gly^338^, Phe^341^, Val^343^, Leu^344^, Leu^345^, Ser^438^, Ser^439^, Gly^442^, Glu^493^, Thr^497^, and Val^501^. The simulation was conducted with the following settings: number of conformations = 50, exhaustiveness = 33, and seed = 2009. The box dimensions were set to XYZ = 30 Å, with central coordinates at X: 169.872, Y: 182.12, and Z: 2.346.

For the minor constituents, including Calarene, γ-himachalene, (±)-cadinene, trans-3-caren-2-ol, humulene/α-caryophyllene, β-guaiene, santolina triene, humulene V1, γ-muurolene, *p*-cymene, phenylephrine, and (1*S*,2*R*)-(+)-norephedrine, molecular docking simulations were performed using the *PyRx* software platform, which integrates *AutoDock Vina* as the docking engine. The same SERT structure was employed to ensure methodological consistency between major and minor constituents. Ligands were energy-minimized within PyRx and converted to the PDBQT format prior to docking. The docking grid was defined to cover the canonical ligand-binding site of SERT, corresponding to the citalopram binding region. For each ligand–protein complex, a total of 20 independent docking runs were conducted, and the binding pose with the lowest predicted binding free energy (Δ*G*) was selected for subsequent interaction analysis.

The molecular dynamics simulation study between the apo SERT transporter and the caryophyllene V1 (CPV1) ligand was conducted using the YASARA Dynamics software package,^37^ parametrized with the AMBER14.^38^ System preparation involved automatic embedding of the SERT in APO state and receptor–ligand complex (SERT::CPV1) into a phospholipid bilayer, using the built-in *md_runmembranefast*.*mcr* script. Helices were used to determine membrane-spanning regions, which were subsequently oriented perpendicular to the membrane plane. A membrane patch of 79 × 79 Å^2^, composed of 100% phosphatidylethanolamine (PEA), was generated and compressed to 75 × 75 Å^2^ to eliminate gaps between lipids. A short MD equilibration run of the compressed membrane was performed to ensure structural continuity.

Water was added using the Truncated/Transferable Intermolecular Potential 3-Point (TIP3P) model, and the system was neutralized to a density of 0.998 g/mL. Ligand CPV1 was parametrized using Austin Model 1 with Bond Charge Corrections (AM1-BCC) and the General AMBER (Assisted Model Building with Energy Refinement) force field 2 (GAFF2), while the protein was handled with AMBER14 parameters. Initial minimization steps included a steepest descent protocol without electrostatics, followed by electrostatics-inclusive minimization. Predicted p*K*_a_ shifts were calculated to adjust protonation states at pH 7.4. Solvent adaptation was refined via simulated annealing and short solvent MD steps, followed by an additional simulated annealing cycle. A final equilibration run of 250 ps was performed, during which water intrusion into the bilayer was restricted. Production MD simulations were then run in duplicate for each ligand–receptor complex, each for 100 ns, under isothermal–isobaric (NPT) conditions (298 K, 2 bar, 0.9% NaCl, with multitime step integration set to 2.5 fs (bonded) and 5.0 fs (nonbonded)). Trajectory analysis was conducted using the *md_analyze*.*mcr* script, which provided quantitative outputs on: Root-mean-square deviation (RMSD); Radius of gyration (*R*_g_); Root-mean-square fluctuation (RMSF); Hydrogen bonding (H-bond). Intermolecular interactions were further characterized with the Arpeggio web server.

### 2- and 3-Dimensional Schematic Representations of the Ligand::Receptor Complex

All docking and molecular dynamics simulations were analyzed using the Molecular Operating Environment (MOE) package, version 2019.0102, to generate 2D representations. The PyMOL Molecular Graphics System, version 1.7.4 (Schrödinger, LLC), and Discovery Studio software (https://discover.3ds.com/discovery-studio-visualizer-download) were used for 3D visualizations. Finally, the types of chemical interactions within the complex were identified using the Arpeggio server.^39^

## Results

### Extraction and EOHc Characterization

#### Oil Yield and Chemical Composition

The oil yield obtained from distillation was 1.0–1.5%. For each 100 g of dried leaves, approximately 300 μL (0.3 mL) of EOHc was obtained. Chromatographic analysis revealed 23 peaks (Figure 2, see Table S1 and Figures S1–S24 in the SI), with 99.99% of the constituents identified. The main constituents are caryophyllene V1 (41.88%), followed by azulene (15.80%), α-pinene (8.89%), bornanone (6.73%), and viridiflorene (4.35%) as shown in Table 1.

**Figure 2:**
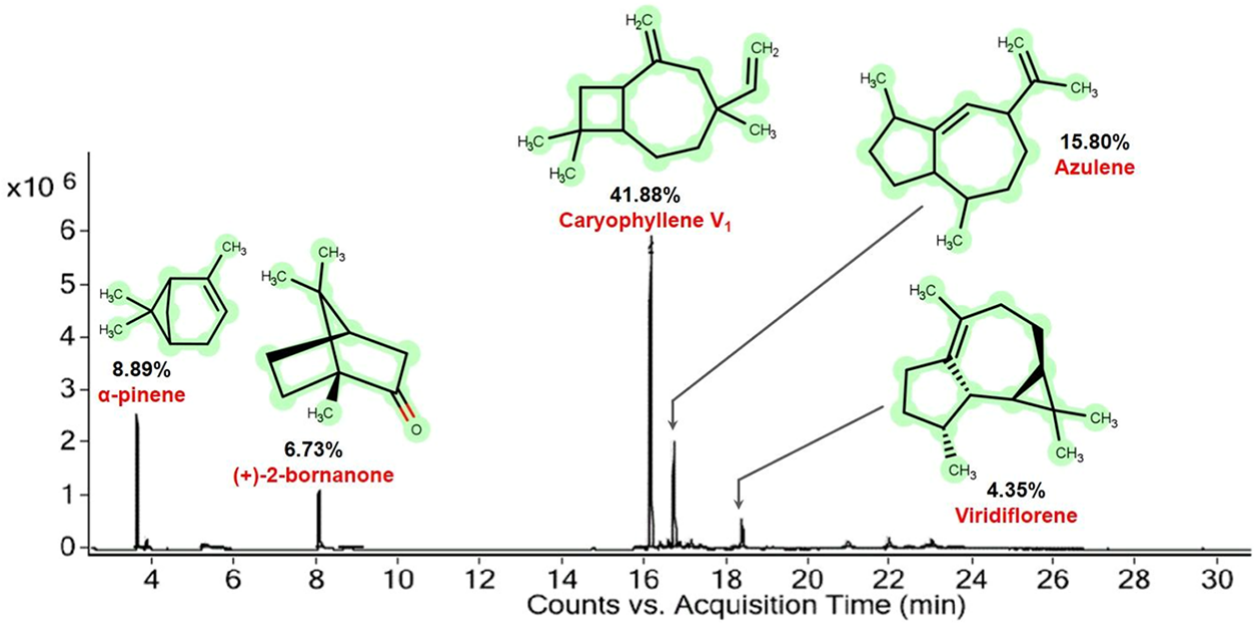
Chromatogram showing the main peaks with the respective EOHc constituents.

**Table 1:** Chemical Constituents of EOHc

identified compound	usual synonym	class	molecular weight	percentage in EOHc
bicyclo[5.2.0]nonane, 2-methylene-4,8,8-trimethyl-4-vinyl	caryophyllene V1	sesquiterpene	204.35	41.88
azulene, 1,2,3,3α,4,5,6,7-octahydro-1,4-dimethyl-7-(1-methylethenyl)-, [1*R*-(1.α.,3 α.β.,4.α.,7.β.)]-	azulene/γ-gurjunene	sesquiterpene	204.35	14.80
(1*R*)-2,6,6-trimethylbicyclo[3.1.1]hept-2-ene	α-pinene	terpene	136.23	8.89
(1*R*,4*R*)-1,7,7-trimethylbicyclo[2.2.1]heptan-2-one	bornanone/δ-camphor	monoterpenoid	152.23	6.73
1*H*-cycloprop[*e*]azulene, 1 α,2,3,5,6,7,7 α,7β-octahydro-1,1,4,7-tetramethyl-, [1 α *R*-(1α.α.,7.α.,7α.β.,7β.α.)]-	(+)-ledene/viridiflorene	sesquiterpene	204.35	4.35
1*H*-cyclopropa[α]naphthalene, 1α,2,3,5,6,7,7α,7β-octahydro-1,1,7,7α-tetramethyl-, [1αR-(1α.α.,7.α.,7α.α.,7β.α.)]-	calarene	sesquiterpenoid	204.35	4.08
4,7,10,13,16,19-docosahexaenoic acid, methyl ester, (all-Z)-		docosahexaenoic acid	792.1	2.66
2,5,9,9-tetramethyl-3,4,4α,7,8,9α-hexahydrobenzo[7]annulene	γ-himachalene	sesquiterpenoid	204.35	2.59
naphthalene, 1,2,4α,5,8,8α-hexahydro-4,7-dimethyl-1-(1-methylethyl)-, (1.α.,4α.β.,8α.α.)-(.±.)-	(±)-cadinene	sesquiterpenoid	204.35	2.49
3,7,7-trimethylbicyclo[4.1.0]hept-3-en-2-ol	*trans*-3-caren-2-ol	monoterpenoid	152.23	2.38
(1*E*,4*E*,8*E*)-2,6,6,9-tetramethylcycloundeca-1,4,8-triene	humulene/α-caryophyllene	sesquiterpene	204.35	1.67
(1*S*,4*S*)-1,4-dimethyl-7-propan-2-ylidene-2,3,4,5,6,8-hexahydro-1H-azulene	β-guaiene	sesquiterpenoid	204.35	1.54
3-ethenyl-2,5-dimethylhexa-1,4-diene	santolina triene	branched unsaturated hydrocarbons	136.23	1.08
1*R*,3*Z*,9s-4,11,11-trimethyl-8-methylenebicyclo[7.2.0]undec-3-ene	humulen V1	sesquiterpene	204.35	0.88
(1*R*,4α*R*,8α*S*)-7-methyl-4-methylidene-1-propan-2-yl-2,3,4α,5,6,8α-hexahydro-1H-naphthalene	γ-muurolene	sesquiterpenoid	204.35	0.68
1-methyl-4-propan-2-ylbenzene	*para*-cymene	monoterpene	134.22	0.64
3-[(1*R*)-1-hydroxy-2-(methylamino)ethyl]phenol	phenylephrine		167.2	0.61
(*S*)-(+)-1-cyclohexylethylamine			127.23	0.57
benzenemethanol,. α.-(1-aminoethyl)-	(1*S*,2*R*)-(+)-norephedrine		151.21	0.47

#### Water and Feed Consumption Weight Monitoring

The average body weight of the mice (Figure 3) did not present significant differences between groups during 7 days of monitoring. The 100 mg treated group of EOHC presented average weight of 30.79 ± 0.27 g, a similar value observed in the 300 mg treated group (30.71 ± 0.39 g) and the group Tween (30.43 ± 0.53 g). Similarly, there were no significant differences between groups and monitoring water consumption and food. The average feed consumption was similar among the groups treated 100 mg (8.03 ± 0.48), 300 mg (7.64 ± 0.65) and Tween (8.04 ± 0.11). The groups that had their water consumption monitored also had similar average water intake.

**Figure 3:**
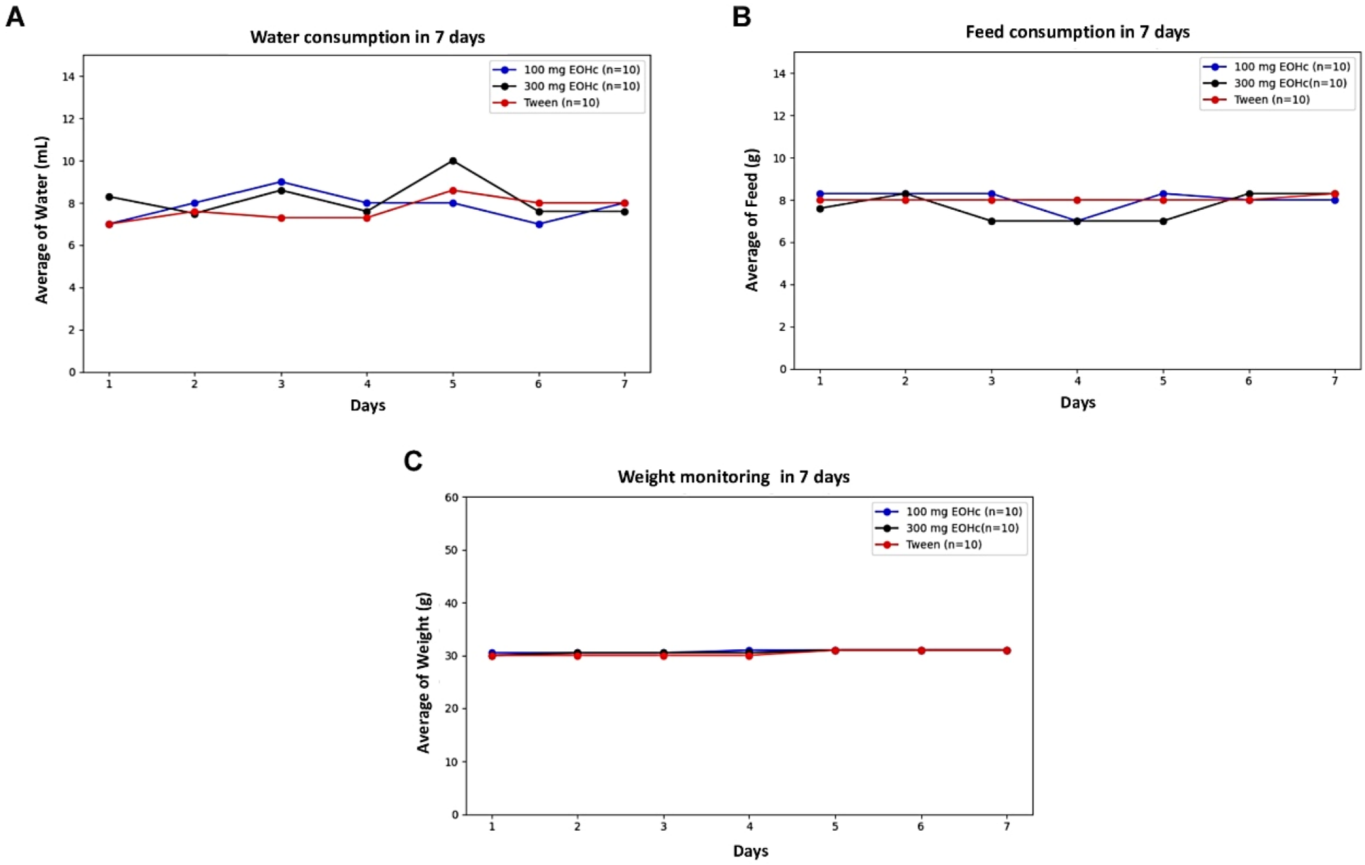
Water and feed consumption weight monitoring: Mice were treated with 100 mg EOHc (blue line), 300 mg EOHc (black line), or Tween (control, red line). A: Average of water consumption in milliliter (mL) per day. B: Average of feed consumption in grams (g) per day. C: Average of weight monitoring in grams (g) per day.

### Behavioral Studies

#### Open Field Test

In the OFT (Figure 4) (*p* > 0.05; *post hoc* ANOVA followed by the Tukey test), the untreated groups after the induction of chronic stress increased the number of crossings (Figure 4A). The groups treated with 100 mg (93.63 ± 11.12) and 300 mg (63.25 ± 10.61) significantly reversed compared to the stressor group (163,64 ± 6.84) (One-way ANOVA F(9.63) = 6.43, *p* < 0.001), as did the citalopram (138.88 ± 11.64) and mirtazapine (182.43 ± 10.81) groups. Regarding the number of rearings (Figure 4B), there was a significant decrease in the groups treated with 100 mg and 300 mg of EOHc (69.00 ± 9.63 and 71.86 ± 5.27) when compared to the stress group (107.88 ± 8.05) (F(9.77) = 5.17; *p* < 0,001). Statistics (*p* > 0.05; *post hoc* ANOVA followed by the Tukey test) also showed that there was no significant difference (F(9.94) = 0.46; *p* = 0.90) between the groups in the grooming count (Figure 4C).

**Figure 4:**
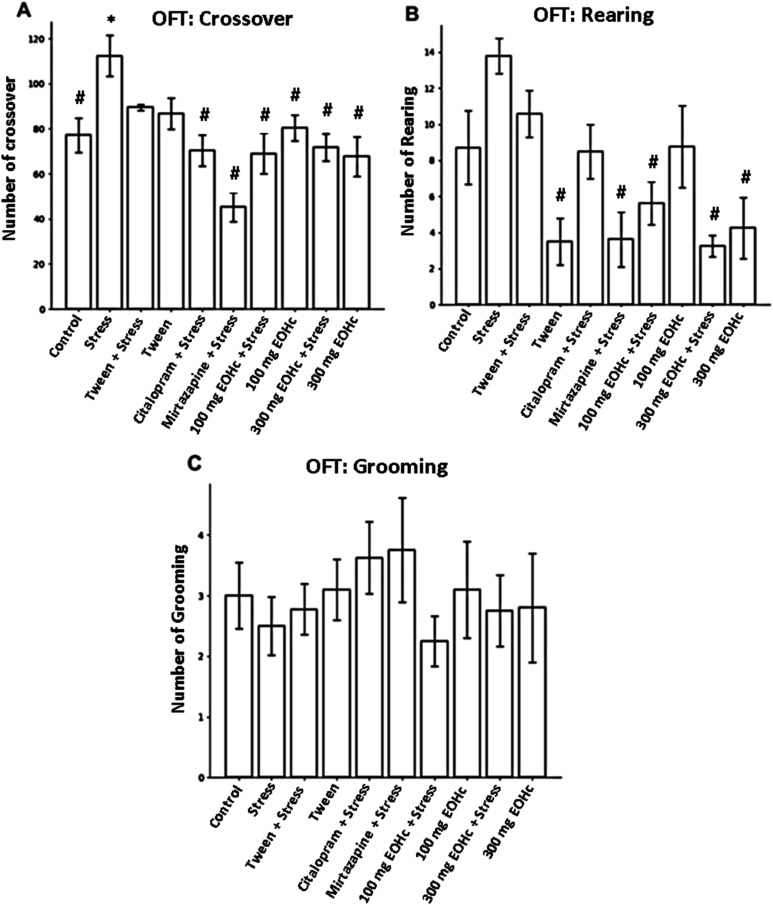
Open Field Test (OFT). (A) Number of crossings in the open field. (B) Rearing count for 5 min. (C) Grooming count for 5 min. Symbol description - # when significant with the stress group; * when significant with the control group.

### Elevated Plus Maze Test

As shown in Figure 5, the stress group (6.55 ± 0.79) and the tween group exposed to chronic stress decreased the number of entries (Figure 5A) and the time (Figure 5C) spent in the open arm (*p* > 0.05; *post hoc* ANOVA followed by the Tukey test). With the exception of the mitazapine group (4.25 ± 0.91 and 53.63 ± 12.15), all groups reverted positively to the control level (F­(9,88) = 13,28; *p* < 0,0001 and F­(9,94) = 16,06; *p* < 0,0001). The number of entries (Figure 5B) into the closed arm did not show a significant difference between the groups, except for the group treated with 300 mg of EOHc (5.0 ± 0.75). The groups treated with 100 mg (93.63 ± 11.12) and 300 mg (63.25 ± 10.61) of EOHc also significantly decreased the time spent in the closed arm (Figure 5D) when compared with the stress group and the chronically stressed tween group (*p* > 0.05; *post hoc* ANOVA followed by the Tukey test).

**Figure 5:**
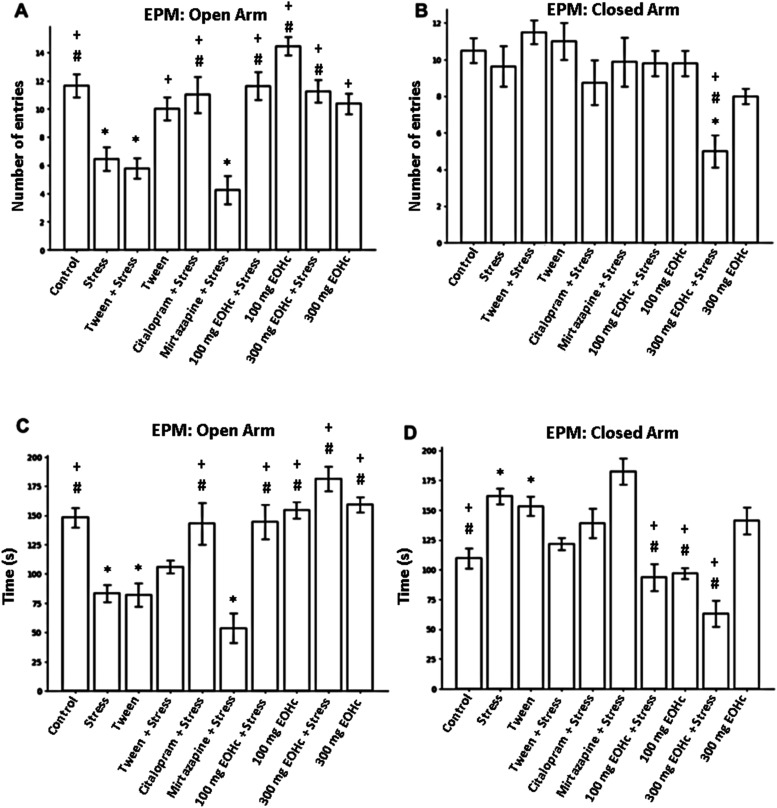
Elevated Plus Maze test. (A) Number of entries of mice into the open arm. (B) Number of entries of mice into the closed arm. (C) Time spent in the open arm in seconds. (D) Time spent in the closed arm in seconds. Symbol description - # when significant with the stress group; + when significant with the stressed Tween group; * when significant with the control group.

### RotaRod Test (RT)

The control group remained in RT for an average of 300.0 ± 0.0 s, while the animals treated with 100 mg (291.0 ± 22.05 s) and 300 mg (300.0 ± 0.0 s) did not have a significant difference from the control group (*p* > 0.05; ANOVA followed by the Tukey test). With a significant difference when compared to control groups, 100 mg and 300 mg (*p* < 0.001). No differences were observed between the control groups, 100 mg and 300 mg (*p* > 0.05).

### *In Silico* Insights into the Main Components of EOHc and apo SERT

#### Bioactivity Prediction Main Components of EOHc

The *in silico* prediction of physicochemical properties for the five major constituents of EOHc (monoterpenes: α-pinene and bornanone; sesquiterpenes: caryophyllene V1, viridiflorene, and azulene) indicates that all compounds comply with Lipinski’s rule of five. All molecules with a molecular weight below 500 Da, a partition coefficient (logP) under 5, fewer than 5 hydrogen bond donors, and fewer than 10 hydrogen bond acceptors. Furthermore, all molecules demonstrated intestinal absorption above 90%, supporting their potential for oral administration. None of the compounds acted as substrates or inhibitors of the primary human cytochrome P_450_ isoforms CYP_3A4_ and CYP_2D6_. *In silico* predictions also suggest that sesquiterpenes may undergo hepatic epoxidation, enhancing their chemical stability and biological potential. Their high lipophilicity favors passage through biological barriers, with log BB and log PS values indicating effective crossing of the blood-brain barrier, making them suitable for central nervous system activity. Additionally, these compounds’ lipophilic characteristics imply a moderate clearance rate, estimated between 5 and 15 mL/min/kg. Toxicological evaluations grouped monoterpenes in classes 4 and 5, with an average LD_50_ of 2237 mg/kg, while sesquiterpenes were grouped in classes 5 and 6, with an average LD_50_ of 5025 mg/kg. All compounds exhibited a low likelihood of neurotoxicity.

### Molecular Docking

A total of 20 molecular docking simulations were performed for each complex formed by apo SERT and the ligands (azulene, bornanone, caryophyllene V1, viridiflorene, and α-pinene), using the AutoDock Vina software. All ligands were capable to bind at both interaction sites (main and allosteric, Figure 6) identified in apo SERT (PDB ID: 5I75), a serotonin transporter (5-HT). For the minor constituents, a total of 20 simulations were also performed for each complex formed with apo SERT using the PyrX software (Table 2).

**Figure 6:**
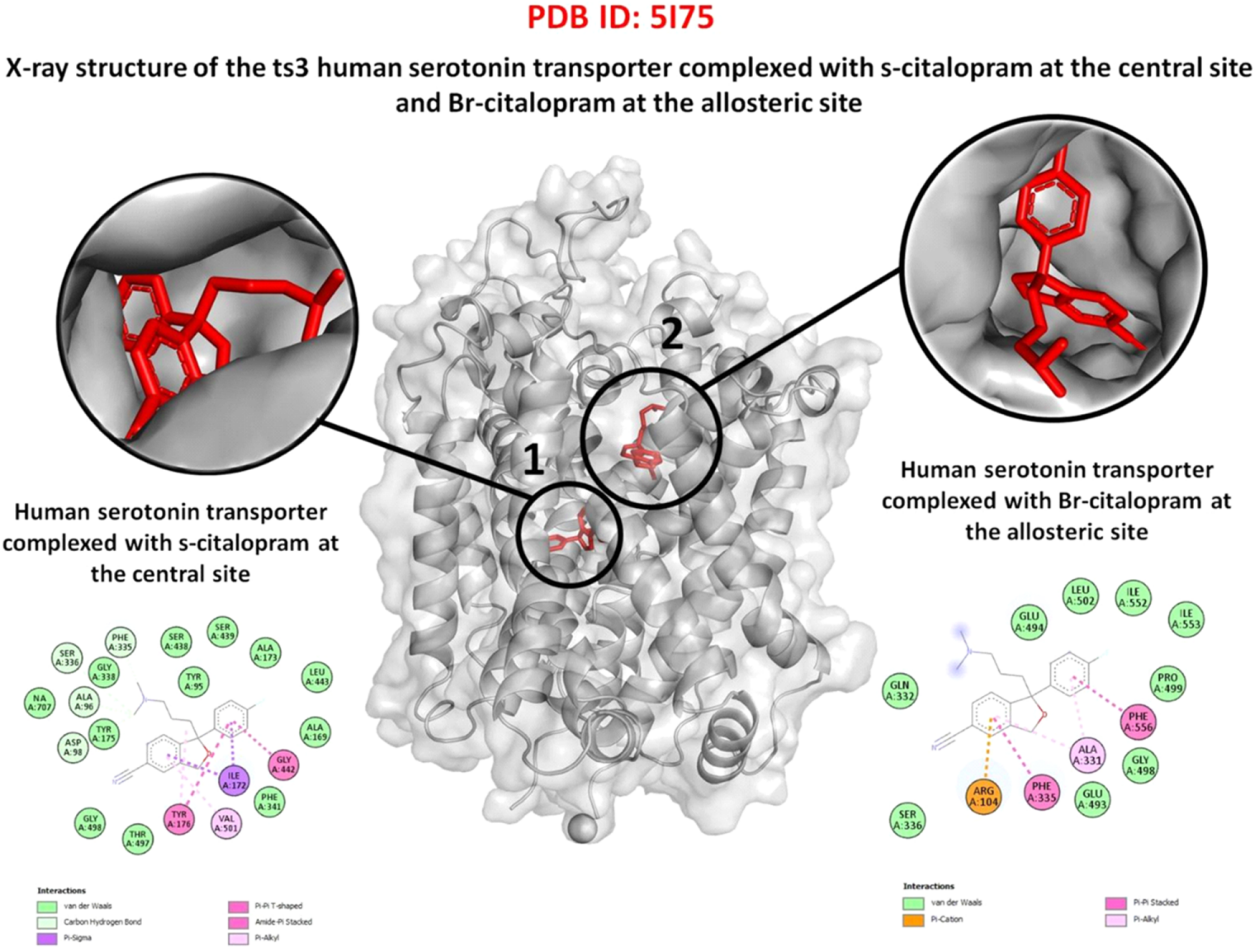
Human serotonin transporter complexed with s-citalopram. Binding at the primary and secondary sites using PDB 5I75 as a guide for studying the serotonin reuptake transporter.

**Table 2:** Molecular Docking Binding Energies (kcal·mol^–1^) of Selected Compounds at the Serotonin Transporter (SERT) Central and Allosteric Binding Sites

**molecule**	**SERT central binding site (kcal mol** ^**–1**^ **)**	**SERT allosteric binding site (kcal mol** ^**–1**^ **)**
cadinene	–7.7	–7.5
calarene	–7.9	–7.9
*p*-cymene	–6.2	–6.1
β-guaiene	–8.3	–7.9
humulene/α-caryophyllene	–8.4	–8.1
humulene (V1)	–8.1	–7.8
(1S,2R)-(+)-norephedrine	–5.8	–5.5
phenylephrine	–5.7	–5.6
santolina triene	–5.5	–5.4
γ-muurolene	–7.8	–7.8
γ-himachalene	–8.3	–8.1
trans-3-carene-2-ol	–6.4	–6.3

The obtained data indicated that the lowest Gibbs free energies (Δ*G*) were observed in the main binding site of apo SERT when complexed with the following ligands: caryophyllene V1 (Δ*G* = −9.8 kcal mol^–1^), azulene (Δ*G* = −9.1 kcal mol^–1^), viridiflorene (Δ*G* = −9.0 kcal mol^–1^), bornanone (Δ*G* = −6.0 kcal mol^–1^), and α-pinene (Δ*G* = −5.8 kcal mol^–1^), respectively (Figure 7A–E).

**Figure 7:**
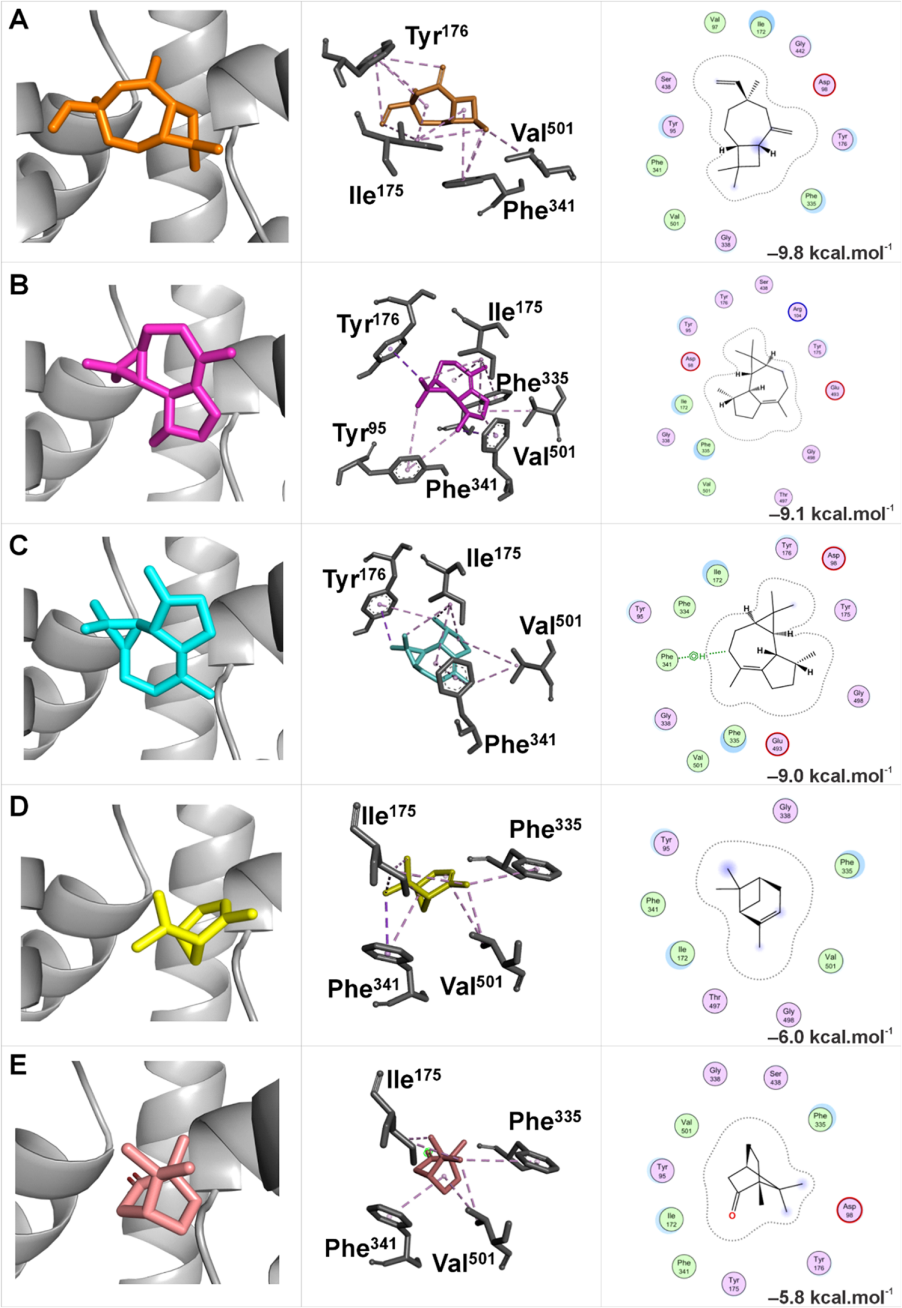
Representation of the molecular docking of the five major compounds from the EOHc at the main binding site of apo SERT. The analyzed compounds include: (A) Caryophyllene V1; (B) Azulene; (C) Viridiflorene; (D) Bornanone; and (E) α-Pinene. The figure is organized into three columns: the first displays the overall visualization of the molecular complex using PyMOL; the second highlights the three-dimensional interactions at the binding site using Discovery Studio; and the third illustrates the two-dimensional interactions, identifying the key residues involved in ligand binding using MOE.

In contrast, the lowest Δ*G* in the allosteric binding site of apo SERT was obtained with the caryophyllene V1 ligand (Δ*G* = −7.8 kcal mol^–1^), followed by azulene (Δ*G* = −7.7 kcal mol^–1^), viridiflorene (Δ*G* = −7.7 kcal mol^–1^), α-pinene (Δ*G* = −6.0 kcal mol^–1^) (Figure 8A–E), and bornanone (Δ*G* = −5.7 kcal mol^–1^), respectively. The Δ*G* values represent the total energy contributions, including van der Waals forces and electrostatic interactions between the apo SERT transporter and the studied ligands.

**Figure 8:**
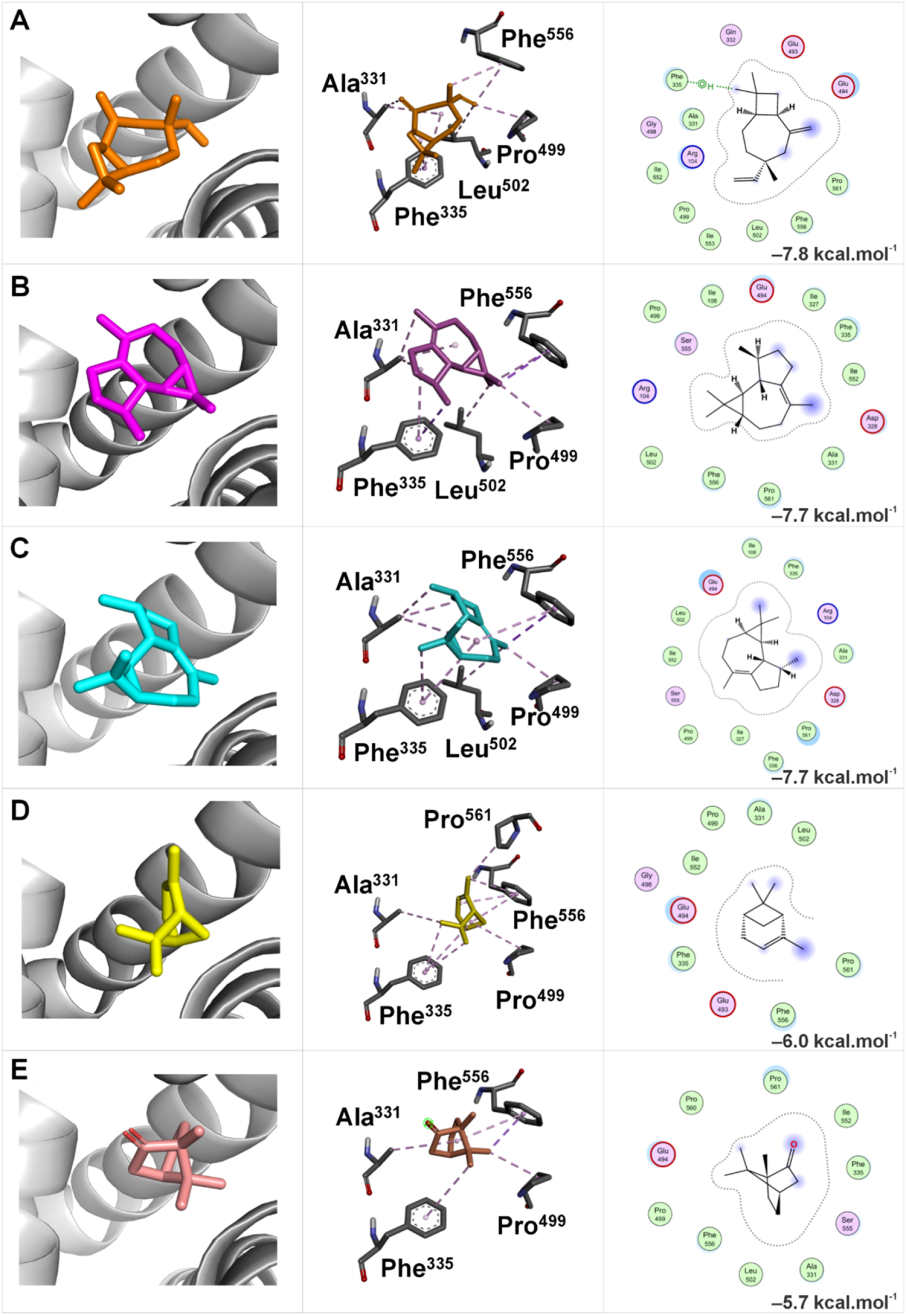
Representation of the molecular docking of the five major compounds from the EOHc at the allosteric binding site of apo SERT. The analyzed compounds include: (A) Caryophyllene V1; (B) Azulene; (C) Viridiflorene; (D) Bornanone; and (E) α-Pinene. The figure is organized into three columns: the first displays the overall visualization of the molecular complex using PyMOL; the second highlights the three-dimensional interactions at the binding site using Discovery Studio; and the third illustrates the two-dimensional interactions, identifying the key residues involved in ligand binding using MOE.

### Molecular Dynamics

Molecular dynamics (MD) analysis demonstrated that some amino acids in the main interaction site of apo SERT were capable of interacting with caryophyllene V1, maintaining a maximum interaction distance of 4.0 Å. After MD simulations, approximately 52.2% of the apo SERT amino acid residues that remained flexible during docking simulations (Tyr^95^, Ala^169^, Ile^172^, Tyr^176^, Ser^336^, Leu^337^, Gly^338^, Phe^341^, Val^343^, Ser^438^, Ser^439^, and Gly^442^) interacted with caryophyllene V1. The MD results indicate that the caryophyllene V1::apo SERT complex is stabilized by a network of charge centers, aromatic rings, hydrophobic interactions, hydrogen bonds, and π-cation interactions (Figure 9, and Table S2 in the SI).

**Figure 9:**
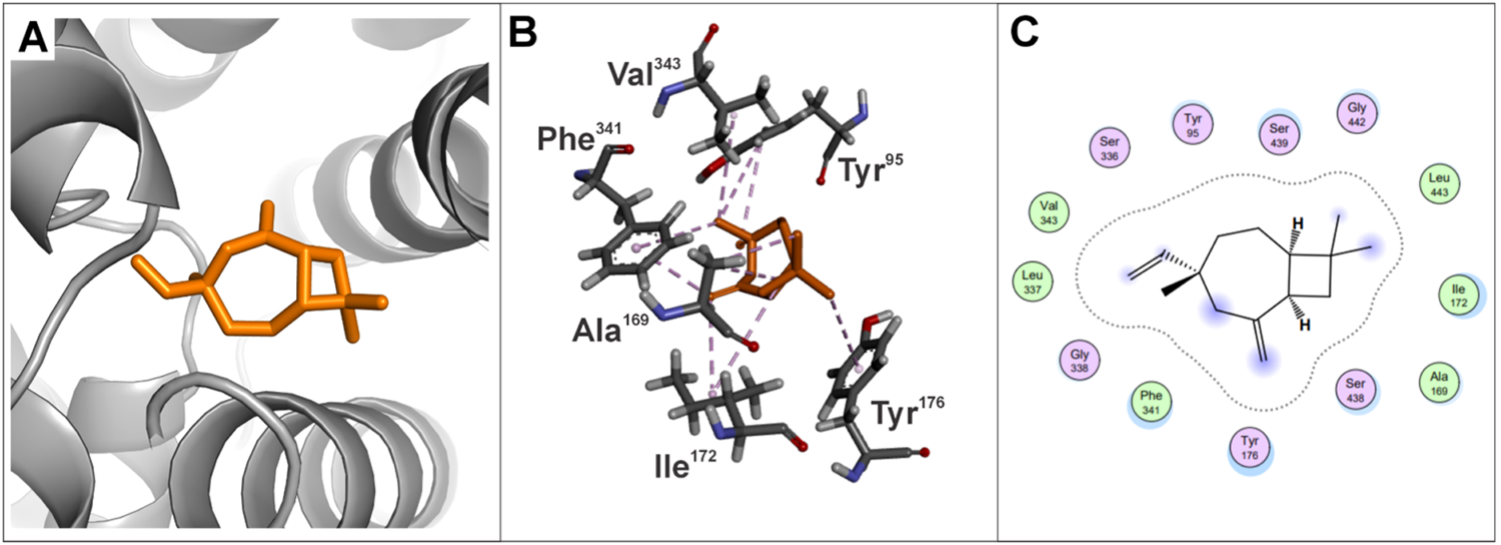
Representation of the interactions following molecular dynamics simulations of caryophyllene V1 at the main binding site of apo SERT. (A) The region demonstrating the greatest stability. (B) 3D visualization of the interactions between the amino acids of the apo SERT and the caryophyllene V1 complex. (C) 2D visualization of the interactions between the amino acids of the apo SERT and the caryophyllene V1 complex. The figure is organized into three columns: the first displays the overall visualization of the molecular complex using PyMOL; the second highlights the 3D interactions at the binding site using Discovery Studio; and the third illustrates the 2D interactions, identifying the key residues involved in ligand binding using MOE.

The Figure 10 compares the root-mean-square deviation (RMSD), radius of gyration (*R*_g_), root-mean-square fluctuation (RMSF), and the number of hydrogen bonds (H-bonds) for apo SERT in its unbound form and in complex with caryophyllene V1. These results suggest that apo SERT has high affinity for caryophyllene V1, as indicated by the low and stable RMSD values (∼1.0 Å) observed throughout the entire simulation time (50 ns). Interestingly, the presence of the ligand (caryophyllene V1) stabilized the conformation of apo SERT, likely due to specific interactions between the ligand and the protein, which restricted its movements (Figure 10A, blue line). Additionally, the MD results are consistent with the molecular docking findings, which previously suggested a favorable interaction between apo SERT and caryophyllene V1, with binding energies (Δ*G*) of −9.8 kcal mol^–1^.

**Figure 10:**
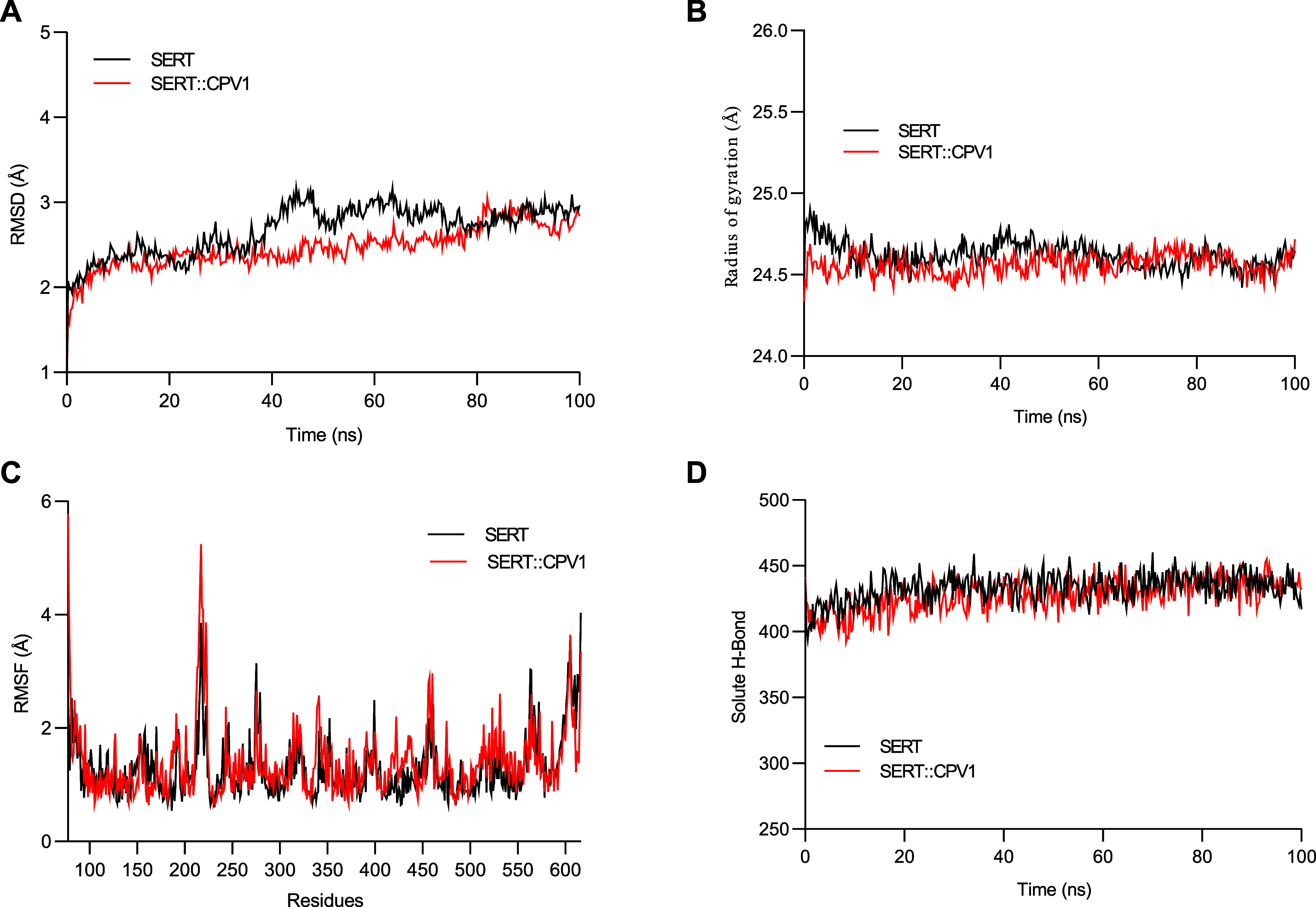
Comparative analysis of molecular dynamics parameters for apo SERT and the SERT::caryophyllene V1 (CPV1) complex over 100 ns of simulation. (A) Root-mean-square deviation (RMSD) showing the structural stability of apo SERT (black line) and the SERT::CPV1 complex (red line). (B) Radius of gyration (*R*_g_) indicating the compactness and structural equilibrium of apo SERT (black line) and the SERT::CPV1 complex (red line). (C) Root-mean-square fluctuation (RMSF) per residue, highlighting local flexibility in apo SERT (black line) and the SERT::CPV1 complex (red line). (D) Number of hydrogen bonds (H-bonds) over simulation time for apo SERT (black line) and the SERT::CPV1 complex (red line), reflecting the stability of interactions. The data suggest that the presence of CPV1 stabilizes the conformation of apo SERT, inducing greater compactness, reducing flexibility in specific regions, and maintaining consistent hydrogen bonding throughout the simulation.

The variation in *R*_g_ over the simulation time for the apo SERT::caryophyllene V1 complex displayed a profile indicative of a more compact and less flexible folding (∼1.0 Å, blue line) compared to apo SERT alone (Figure 10B). The data suggest that the presence of caryophyllene V1 induces slightly lower *R*_g_ values at certain points, indicating increased compactness. This compactness is accompanied by greater structural equilibrium, as evidenced by the minimal fluctuations in the radius of gyration. Similar to the RMSD results, the *R*_g_ data suggest that caryophyllene V1 contributes to the compactness and stabilization of apo SERT in the complex.

The RMSF variation as a function of simulation time also demonstrates that the apo SERT::caryophyllene V1 complex exhibits a more stable profile than apo SERT alone (Figure 10C). This is evident from the lower peaks in RMSF, indicating that residues or regions of apo SERT they are less flexible (blue line). The fluctuations are smaller in several regions, particularly in residues directly interacting with caryophyllene V1. Thus, the presence of the ligand reduces the flexibility of specific regions of apo SERT, indicating direct interactions and conformational stabilization.

The number of hydrogen bonds (Figure 10D) remains comparable between apo SERT (red line) and the apo SERT::caryophyllene V1 complex (blue line). While minor fluctuations are observed, the overall values are consistent, suggesting that the system remains stable throughout the simulation. Although the ligand stabilizes the complex, caryophyllene V1 does not appear to induce significant changes in the global hydrogen bonding pattern of apo SERT. Together, these findings suggest that apo SERT is more stable in the presence of caryophyllene V1, as evidenced by consistently stable interactions and reduced flexibility.

## Discussion

The present study provides convergent behavioral, pharmacological, and molecular evidence supporting the anxiolytic-like activity of *H. crenata* essential oil (EOHc), offering a comprehensive mechanistic framework that aligns more closely with serotonergic reuptake modulation than with receptor-level antagonism. From a translational standpoint, this distinction is particularly relevant, as it informs not only the interpretation of behavioral outcomes but also the comparative efficacy observed among the reference pharmacological agents employed in this study, especially within experimental paradigms sensitive to serotonergic tone.

In this context, it is noteworthy that this study demonstrates, for the first time, that EOHc exerts anxiolytic-like effects in mice, supported by consistent and complementary behavioral, molecular docking, and molecular dynamics findings. Molecular docking analyses revealed that key EOHc constituents, including the monoterpenes α-pinene and bornanone, as well as the sesquiterpenes caryophyllene V1, viridiflorene, and azulene — bind to sites within the serotonin transporter (SERT) that overlap with, or are proximal to, those targeted by selective serotonin reuptake inhibitors (SSRIs), such as citalopram, which was used as the positive control in this study. These observations suggest that the anxiolytic-like effects of EOHc may be associated with serotonergic modulation mediated by its terpenoid components, rather than through indirect monoaminergic mechanisms.

From a safety and tolerability perspective, no significant alterations were observed in body weight, water intake, or food consumption during the 7-day treatment period with EOHc at doses of 100 and 300 mg/kg. These findings are consistent with previous reports demonstrating the safety of this essential oil at doses up to 2000 mg/kg.^20^ Such observations are particularly relevant when considering the translational potential of phytochemical-based interventions, as systemic toxicity and metabolic disturbances often represent limiting factors in preclinical development.

Behavioral assessments further corroborated the anxiolytic-like profile of EOHc. In the OFT, a classical paradigm used to assess exploratory behavior and emotional reactivity,^40,41^ EOHc treatment resulted in reduced locomotor activity (crossings) and rearing behavior. The decrease in locomotor activity in OFT is frequently associated with the sedative effect.^42,43^ Importantly, this behavioral profile closely resembled that observed in animals treated with citalopram, an SSRI widely prescribed for anxiety disorders. The reduction in exploratory drive observed in the OFT, therefore, appears to reflect a modulation of anxiety-related behavior rather than nonspecific motor suppression. This interpretation is further supported by the results of the RT, which demonstrated that EOHc did not impair motor coordination, balance, or motor learning, even at the higher dose tested.^44^ The absence of sedative or motor-impairing effects is critical for excluding confounding factors that could artificially influence exploratory behavior in anxiety-related paradigms.

Regarding the OFT, grooming can be used to assess the relevant participation of dopaminergic pathways, related to the significant increase in this stereotypical behavior.^45^ Thus, the analysis of grooming in the OFT contributes as an important complementary marker for understanding the emotional state and behavioral reactivity of mice.^46^ The absence of significance in this parameter suggests that treatment with EOHc did not promote relevant changes in the emotional state associated with stress or in stereotyped mechanisms that could be linked to dopaminergic hyperactivity.

While the OFT provides valuable preliminary insights into emotional and exploratory behavior, its specificity for anxiolytic screening is inherently limited.^47^ For this reason, the EPM, a more specific and extensively used paradigm for evaluating anxiety-like behavior,^48,49^ was employed to further substantiate the behavioral findings. In this paradigm, EOHc at both 100 and 300 mg/kg significantly increased both the number of entries into, and the time spent in, the open arms, classical indices of reduced anxiety. These effects closely paralleled those observed with citalopram,^50^ thereby reinforcing the anxiolytic-like potential of EOHc and suggesting convergence at the level of serotonergic modulation.

A particularly informative aspect of the behavioral data emerges when considering the differential performance of mirtazapine compared to citalopram and EOHc, especially in the EPM. Mirtazapine exerts its antidepressant and anxiolytic effects primarily through antagonism of presynaptic α2-adrenergic autoreceptors and heteroreceptors, leading to increased noradrenergic and serotonergic release, in combination with selective antagonism at 5-HT2 and 5-HT3 receptors.^51,52^ Furthermore, mirtazapine also exerts a mechanism of action through antagonism of H1 histamine receptors.^53^ The unpredictable stress model was effective; however, mirtazapine did not show an anxiolytic effect in this experimental context. Although the treatment was carried out for 7 days, this period is considered subchronic and may be insufficient for the full development of the anxiolytic effects of mirtazapine, which generally require prolonged chronic administration.^54,55^ Thus, the effects observed in the OFT and EPM may predominantly reflect initial pharmacological actions, such as antagonism of H1 histamine receptors, resulting in decreased exploratory activity, possibly associated with a sedative or hypoactive drug profile. In contrast, the present in silico results strongly suggest that the constituents of EOHc interact directly with SERT, positioning EOHc mechanistically closer to SSRIs than to atypical antidepressants such as mirtazapine.

This mechanistic distinction is further reinforced by the sensitivity of the chronic unpredictable stress (CUS) model employed in this study. Although mirtazapine is clinically effective in anxiety and depressive disorders, its comparatively limited efficacy in reversing anxiety-like behavior in this paradigm suggests that the CUS protocol used here may preferentially detect interventions that directly modulate serotonergic reuptake rather than those that act indirectly via receptor antagonism. Such observations underscore the importance of aligning preclinical models with the specific pharmacodynamic mechanisms under investigation and enhance the interpretability of the behavioral outcomes.^56^

At the molecular level, docking simulations revealed that all major terpenoid constituents of EOHc exhibit affinity for both the primary (orthosteric) and secondary (allosteric) binding sites of apo SERT (PDB ID: 5I75). Caryophyllene V1 displayed the most favorable binding energy (Δ*G* = −9.8 kcal mol^–1^), followed by azulene (−9.1 kcal mol^–1^) and viridiflorene (−9.0 kcal mol^–1^), values comparable to those reported for clinically used SSRIs. Importantly, these findings extend beyond simple ligand–target association, as molecular dynamics simulations demonstrated sustained and stable interactions of caryophyllene V1 with key SERT residues over time. These interactions were accompanied by reductions in root-mean-square deviation (RMSD), radius of gyration (*R*_g_), and residue-level fluctuations (RMSF), indicative of a stabilizing effect on both global and local protein conformations.^57^

The involvement of the SERT allosteric site warrants particular attention, as this region has emerged as a critical modulator of transporter function and pharmacology. Allosteric ligands are known to influence the binding kinetics and dissociation rates of orthosteric inhibitors, potentially enhancing efficacy, prolonging transporter inhibition, or modulating conformational transitions required for substrate translocation. In this context, the ability of caryophyllene V1 and other EOHc terpenoids to interact with both orthosteric and allosteric sites raises the possibility of cooperative or allosterically enhanced inhibition of serotonin reuptake. Such dual-site engagement may confer pharmacological advantages, including reduced required doses, improved selectivity, and a potentially more favorable side-effect profile compared to classical high-affinity orthosteric inhibitors.

From a translational and clinical perspective, this allosteric modulation paradigm is particularly attractive, as it aligns with emerging strategies aimed at fine-tuning neurotransmitter systems rather than producing maximal blockade. The stabilization of specific SERT conformations by terpenoid ligands may allow for partial inhibition of serotonin reuptake, preserving physiological serotonergic signaling while attenuating pathological anxiety-related hyperreactivity. This mechanism may also contribute to improved tolerability, an important consideration in the long term management of anxiety disorders.

Collectively, the integration of behavioral assays, molecular docking, and molecular dynamics simulations provides a coherent and mechanistically grounded interpretation of EOHc’s anxiolytic-like effects. The data strongly support a model in which terpenoid constituents, particularly caryophyllene V1, act as functional modulators of the serotonin transporter, engaging both orthosteric and allosteric sites to stabilize SERT conformations and mimic key aspects of SSRI pharmacology. This multimodal approach not only validates the traditional use of *H. crenata* but also positions EOHc as a promising source of serotonergic modulators with potential translational relevance.

Therefore, future studies incorporating neurochemical assays, transporter kinetics, pharmacokinetic profiling, and clinical-oriented behavioral end points will be essential to further delineate the therapeutic potential and mechanistic nuances of EOHc. Although the stress protocol required adjustments due to ethical constraints, the close correspondence between EOHc and conventional serotonergic treatments strengthens the translational significance of these findings and supports continued investigation into phytochemical-based modulators of serotonergic neurotransmission.

## Conclusion

This study demonstrates the anxiolytic-like effect of EOHc, supported by behavioral tests and molecular docking analysis. EOHc significantly modulated stress-induced behaviors, with effects comparable to citalopram and mirtazapine. Docking results suggest that terpenoids in EOHc, particularly caryophyllene V1, azulene, and viridiflorene, may interact with serotonin transporter sites, resembling serotonin reuptake inhibitors’ mechanism of action. However, we cannot rule out the effect of other constituents present in EOHc in low concentrations. These findings validate EOHc’s therapeutic potential for anxiety.

## Supplementary Material



## Notes

Research approval file in Animal Ethics Committee of Christus University (S1Material); complete technical material of chromatographic analysis revealed 23 peaks (S2Material); interactions table, the caryophyllene V1::apo SERT (S3Material—Table S1) (PDF)
