# Correction to “Anxiolytic-Like Effect of *Hyptis* crenata Essential Oil: Behavioral Insights and *In Silico* SERT Modulation”

**DOI:** 10.1021/acsomega.6c10771

**Published:** 2026-10-09

**Authors:** Savyo Mikael Lacerda Gomes, André Nogueira Cardeal dos Santos, José Lucas Gomes Izidorio, José Ednésio da Cruz Freire, Kirley Marques Canuto, Marília Cavalcante Araújo, Francisco Sydney Henrique Félix, Marcus Vinícius Vieira Torquato, Jonathan Elias Rodrigues Martins, Yuri Abreu Gomes-Vasconcelos, Kerly Shamyra Silva Alves, José Henrique Leal Cardoso, José Eduardo Ribeiro Honório Júnior, Andrelina Noronha Coelho de Souza

Figures 7, 8, and 9 have been replaced to address publication-permissions concerns relating to portions of the originally published figures. The replacement figures remove those portions and provide corrected visualizations. These changes do not affect the results, discussion, or conclusions of the article.

**Figure 7:**
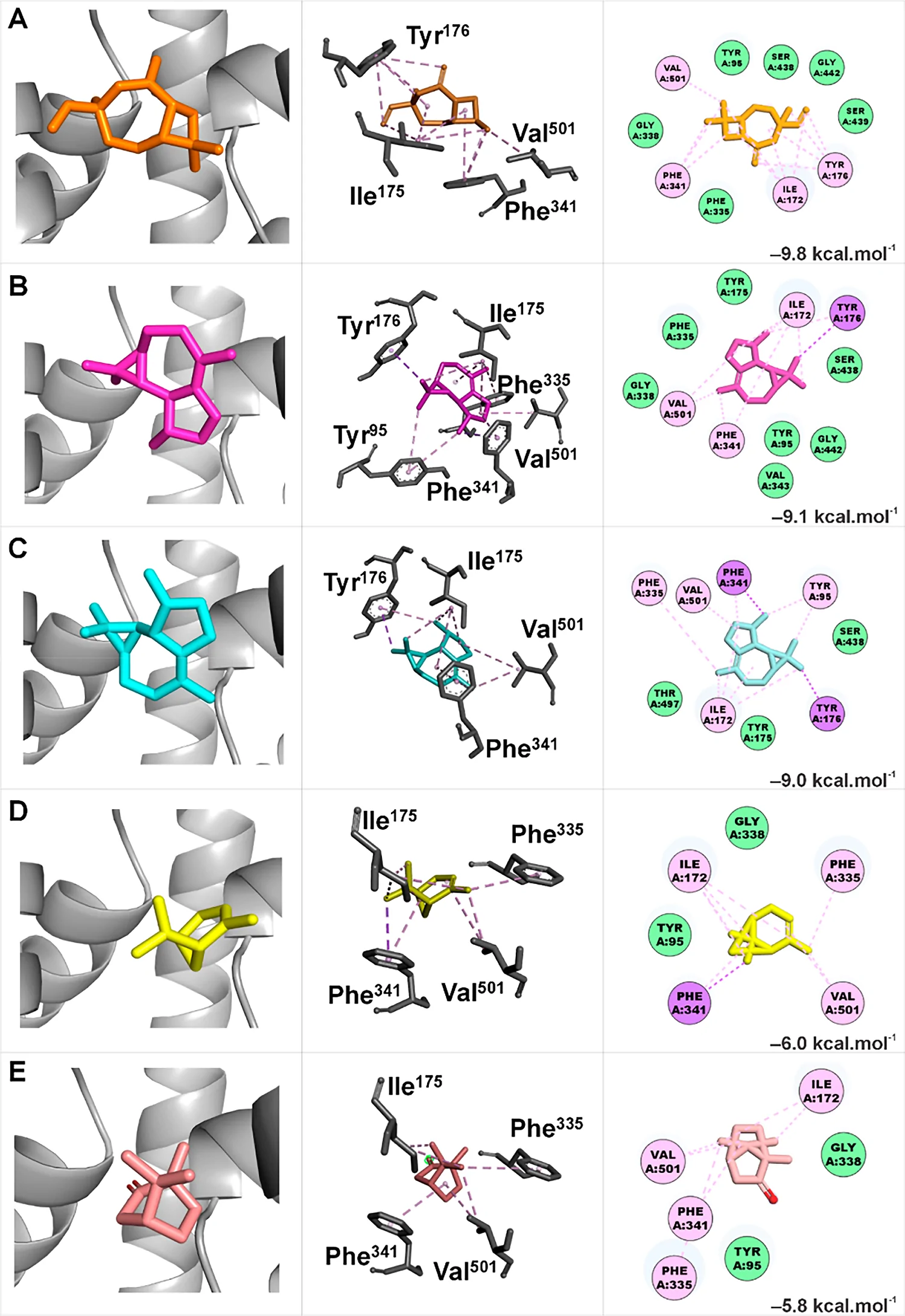
Representation of the molecular docking of the five major compounds from the EOHc at the main binding site of apo SERT. The analyzed compounds include (A) Caryophyllene V1; (B) Azulene; (C) Viridiflorene; (D) Bornanone; and (E) α-Pinene. The figure is organized into three columns: the first displays the overall visualization of the molecular complex using PyMOL; the second highlights the three-dimensional interactions at the binding site using Discovery Studio; and the third illustrates the two-dimensional interactions, identifying the key residues involved in ligand binding using Discovery Studio.

**Figure 8:**
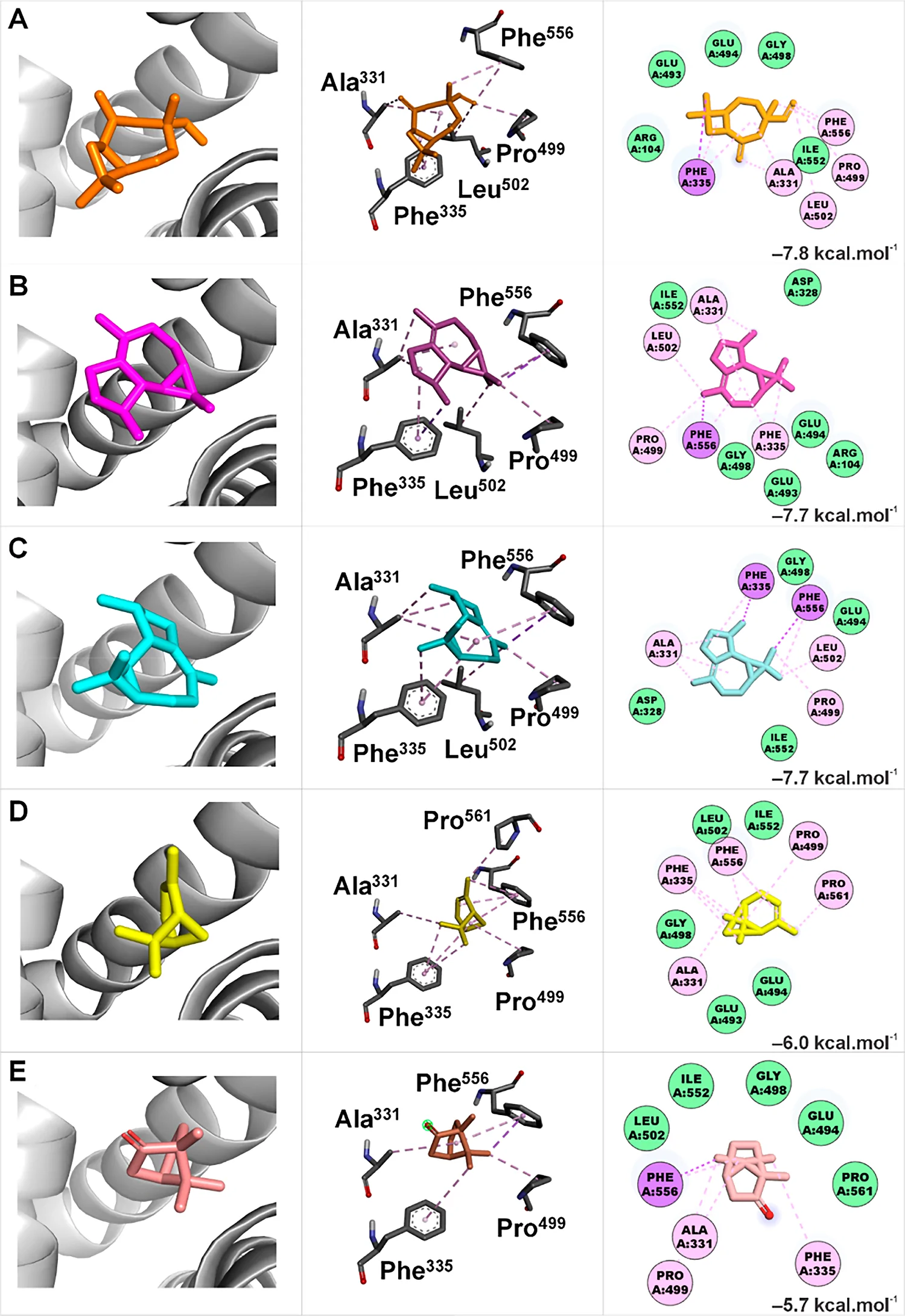
Representation of the molecular docking of the five major compounds from the EOHc at the allosteric binding site of apo SERT. The analyzed compounds include (A) Caryophyllene V1; (B) Azulene; (C) Viridiflorene; (D) Bornanone; and (E) α-Pinene. The figure is organized into three columns: the first displays the overall visualization of the molecular complex using PyMOL; the second highlights the three-dimensional interactions at the binding site using Discovery Studio; and the third illustrates the two-dimensional interactions, identifying the key residues involved in ligand binding using Discovery Studio.

**Figure 9:**
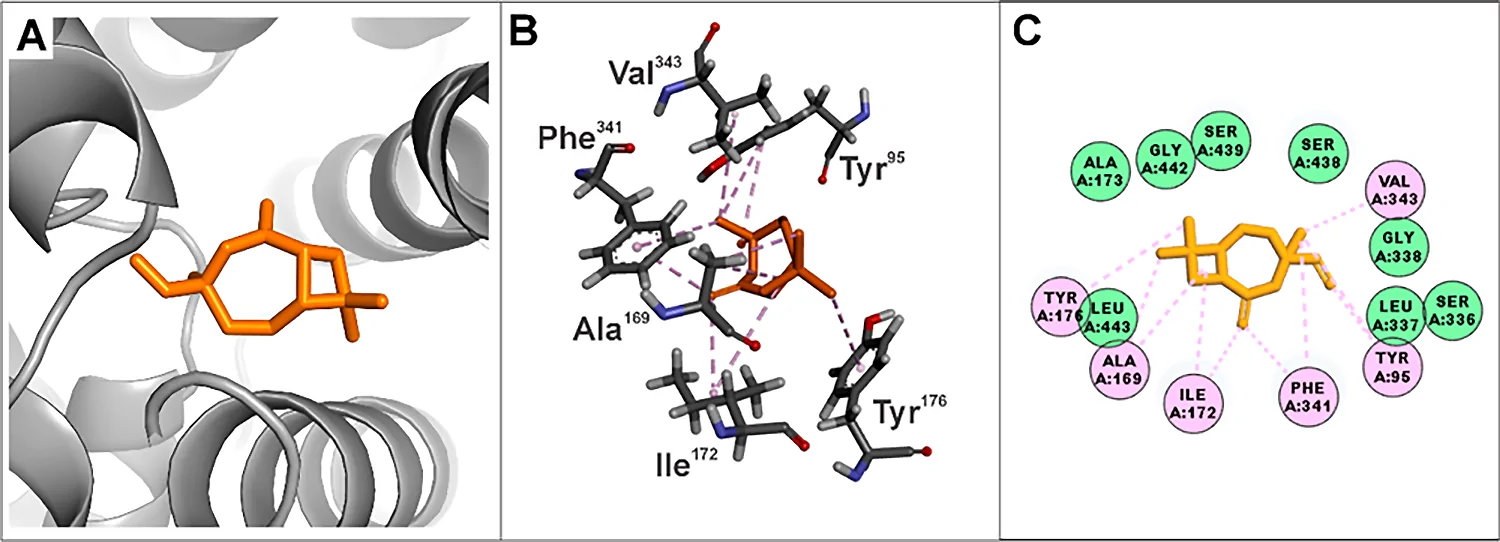
Representation of the interactions following molecular dynamics simulations of caryophyllene V1 at the main binding site of apo SERT. (A) The region demonstrating the greatest stability. (B) 3D visualization of the interactions between the amino acids of the apo SERT and the caryophyllene V1 complex. (C) 2D visualization of the interactions between the amino acids of the apo SERT and the caryophyllene V1 complex. The figure is organized into three columns: the first displays the overall visualization of the molecular complex using PyMOL; the second highlights the 3D interactions at the binding site using Discovery Studio; and the third illustrates the 2D interactions, identifying the key residues involved in ligand binding using Discovery Studio.

