# Supplementary material for: Anxiolytic-Like Effect of Hyptis crenata Essential Oil: Behavioral Insights and In Silico SERT Modulation
Source: ACS Omega. 2026 Mar 6;11(11):17589–604. doi: 10.1021/acsomega.5c11201 (PMC13019207; doi:10.1021/acsomega.5c11201)
Supplement: Supplementary file 1 [file ao5c11201_si_001.pdf]

SUPPORTING INFORMATION

Anxiolytic-like effect of *Hyptis crenata* essential oil: Behavioral insights and *in silico* SERT modulation

Savyo Mikael Lacerda Gomes[a,b], André Nogueira Cardeal dos Santos[a,b*], José Lucas Gomes Izidorio[b], José Ednésio da Cruz Freire[c], Kirley Marques Canuto[d], Marília Cavalcante Araújo[e], Francisco Sydney Henrique Félix[a], Marcus Vinícius Vieira Torquato[a], Jonathan Elias Rodrigues Martins[c], Yuri Abreu Gomes-Vasconcelos[a], Kerly Shamyra Silva Alves[e], José Henrique Leal Cardoso[e], José Eduardo Ribeiro Honório Júnior[b], Andrelina Noronha Coelho de Souza[a*].

[a] *Experimental Physiology Laboratory, State University of Ceará, Superior Institute of Biomedical Sciences, Ave. Dr. Silas Munguba - 1700, 60714-903, Fortaleza, Ceará, Brazil.*

[b] *Neuroscience and Translational Medicine Laboratory, Christus University - Unichristus, St. João Adolfo Gurgel – 133, 60192-345, Fortaleza, Ceará, Brazil.*

[c] *Biochemistry and Gene Expression Laboratory, State University of Ceará, Superior Institute of Biomedical Sciences, Ave. Dr. Silas Munguba - 1700, 60714-903, Fortaleza, Ceará, Brazil.*

[d] *Laboratory of Natural Products Chemistry, Embrapa Tropical Agroindustry, Brazilian Agricultural Research Corporation, St. Doutora Sara Mesquita – 2270, 60511-110, Fortaleza, Ceará, Brazil.*

[e] *Electrophysiology Laboratory, State University of Ceará, Superior Institute of Biomedical Sciences, Ave. Dr. Silas Munguba - 1700, 60714-903, Fortaleza, Ceará, Brazil.*

[*] Email: andrecardealdossantos@gmail.com

[*] Email: andrelina.noronha@uece.br

TABLE S1

Table S1 – GCMS peak list of the essential oil, including Peak, Start, retention times (RT), End, Height, Area, and Area Sum Percent.

Integration Peak List

Peak	Start	RT	End	Height	Area	AreaSumPercent
1	3.571	3.6	3.721	2536434.16	3592151.06	8.89
2	3.791	3.848	3.931	207888.34	437246.17	1.08
3	5.172	5.216	5.254	84163.61	259671.02	0.64
4	5.254	5.286	5.751	105231.29	960152.36	2.38
5	7.997	8.041	8.162	1102184.76	2721058.4	6.73
6	8.588	8.664	9.129	25501.2	230078.09	0.57
7	15.784	15.834	16.032	33415.43	276494.1	0.68
8	16.044	16.121	16.28	5923001.98	16930458.77	41.88
9	16.312	16.369	16.432	147356.01	454416.54	1.12
10	16.522	16.566	16.63	192945.29	555620	1.37
11	16.63	16.693	16.789	2027766.11	5793871.92	14.33
12	16.789	16.827	16.96	162581.51	592336.64	1.47
13	16.96	17.024	17.075	114986.79	356424.2	0.88
14	17.075	17.126	17.279	172851.25	677134.75	1.67
15	17.279	17.349	17.543	95259.89	369159.23	0.91
16	18.11	18.182	18.303	60054.54	274353.33	0.68
17	18.303	18.36	18.513	563767.15	1756903.48	4.35
18	20.841	20.949	21.166	154550.79	1046763.38	2.59
19	21.859	21.936	22.171	215865.41	1008137.06	2.49
20	22.381	22.445	22.623	35921.73	247609.1	0.61
21	22.769	22.871	22.954	96486.8	620556.44	1.54
22	22.954	22.992	23.297	185861.47	1074166.56	2.66
23	23.399	23.456	23.838	18390	191782.95	0.47

FIGURE S1

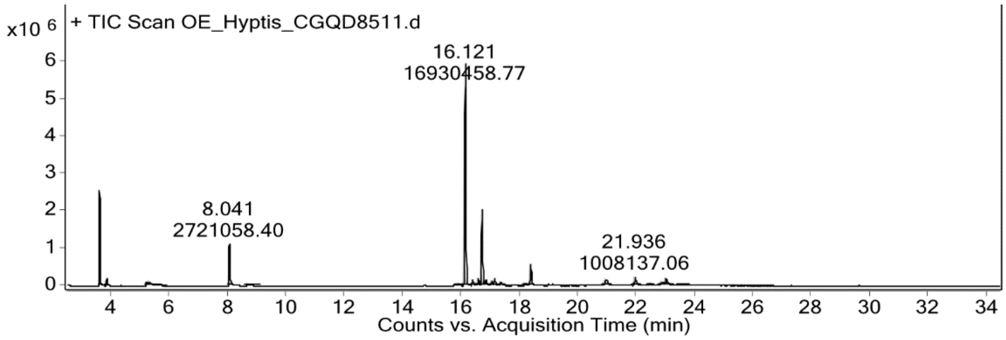

Figure S1 – Total Ion Chromatogram (TIC) of the essential oil from *Hyptis crenata* obtained by GC-MS analysis. The peaks represent the volatile constituents identified in the sample, with major components indicated by their respective retention times (min).

FIGURE S2

A

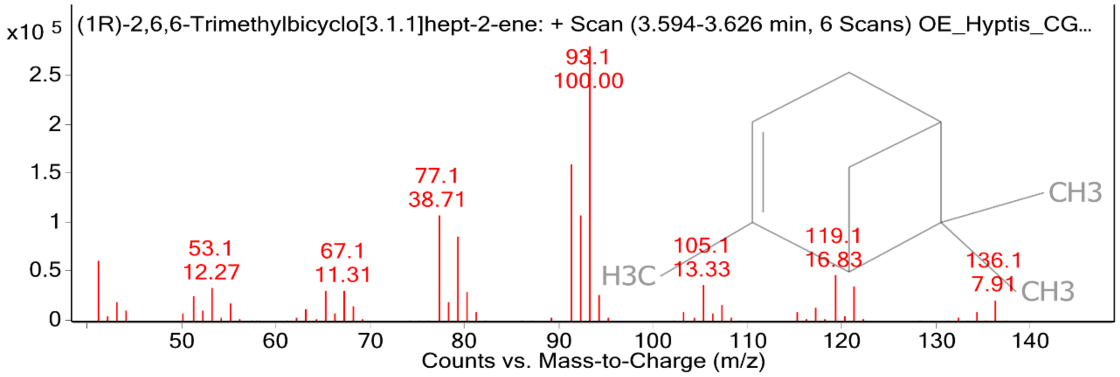

B

Library Spectrum

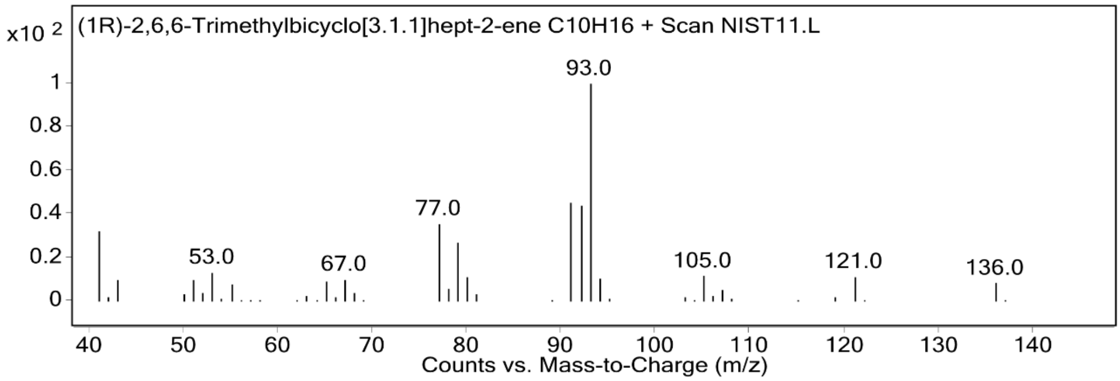

C

Spectrum Structure

(1R)-2,6,6-Trimethylbicyclo[3.1.1]hept-2-ene

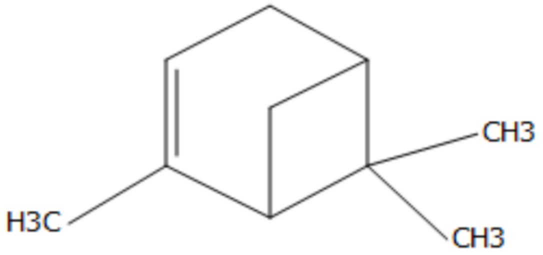

Figure S2 - Mass spectrum of Peak 1 from the TIC chromatogram, assigned to (1R)-2,6,6-Trimethylbicyclo[3.1.1]hept-2-ene (usual synonym: **α-pinene)**. (A) Experimental mass spectrum extracted from the chromatographic analysis. (B) Reference mass spectrum from the NIST11 library used for identification. (C) Chemical structure of the identified compound.

FIGURE S3

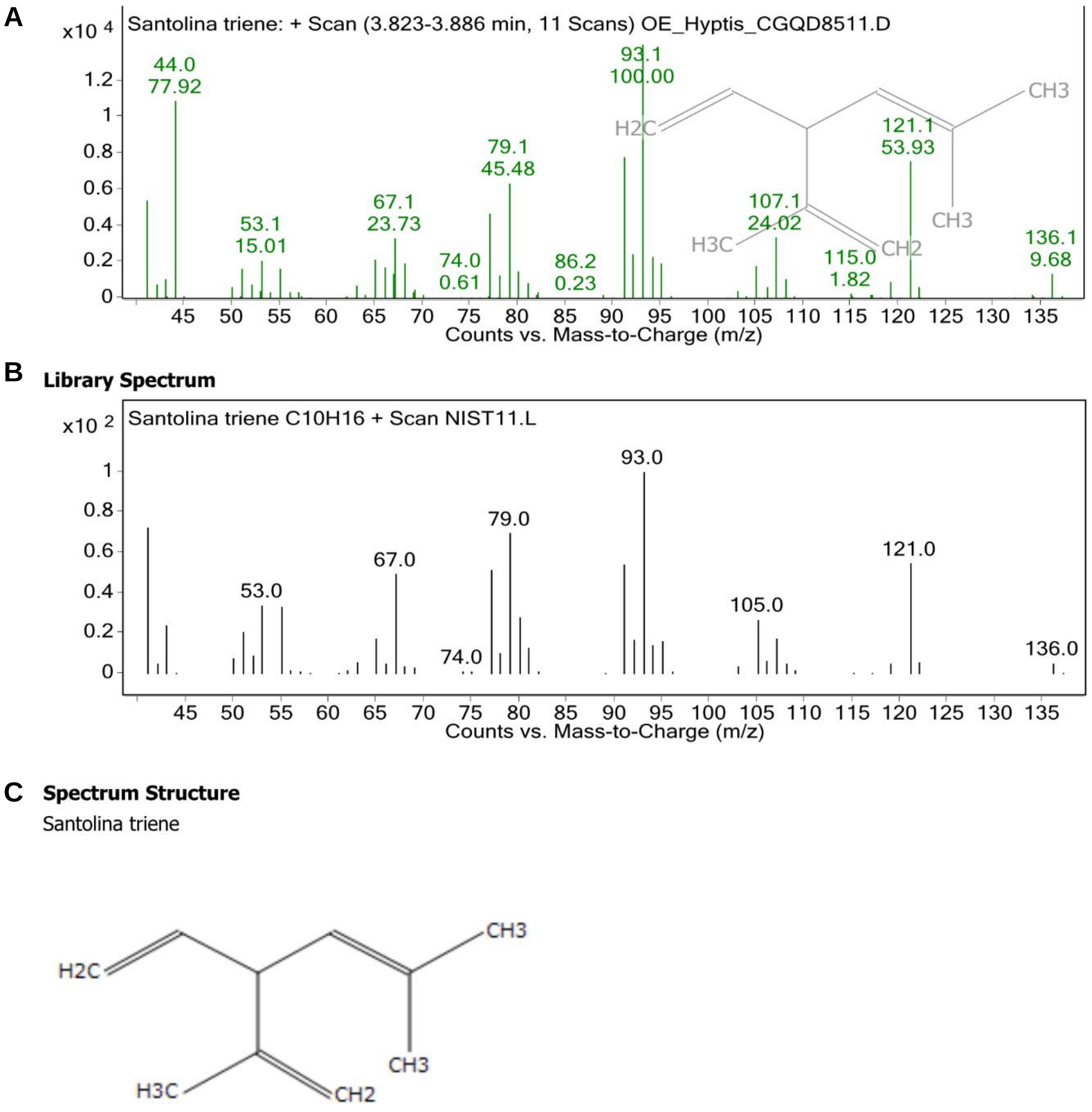

Figure S3 - Mass spectrum of Peak 2 from the TIC chromatogram, assigned to **santolina triene**. (A) Experimental mass spectrum extracted from the chromatographic analysis. (B) Reference mass spectrum from the NIST11 library used for identification. (C) Chemical structure of the identified compound.

FIGURE S4

A

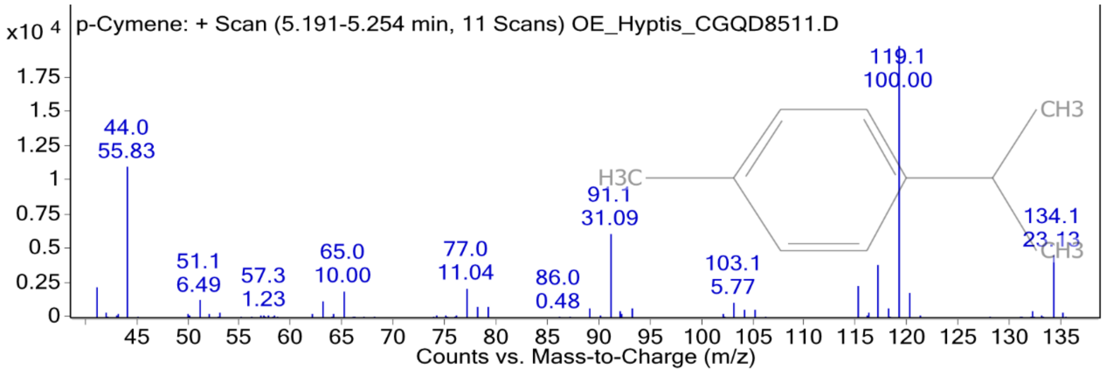

B **Library Spectrum**

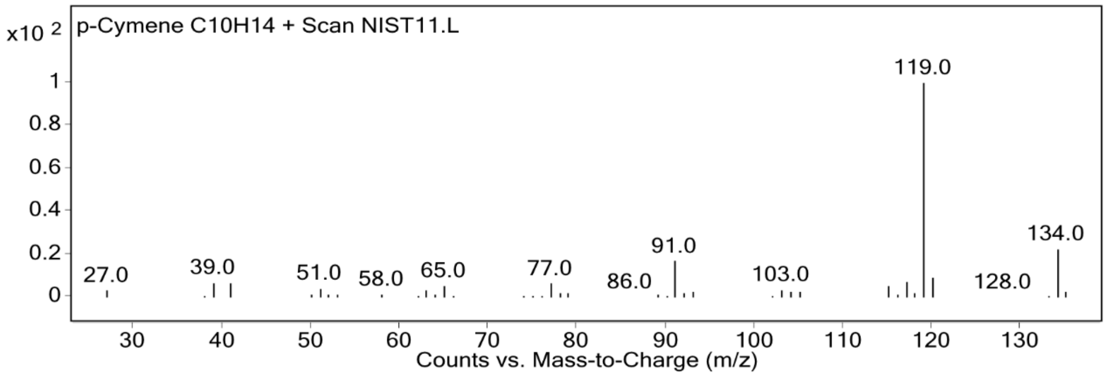

C **Spectrum Structure**

p-Cymene

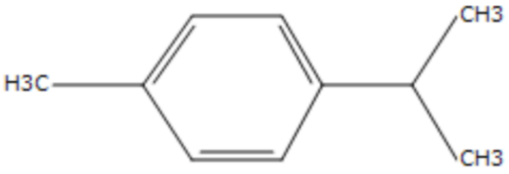

Figure S4 - Mass spectrum of Peak 3 from the TIC chromatogram, assigned to **p-Cymene**. (A) Experimental mass spectrum extracted from the chromatographic analysis. (B) Reference mass spectrum from the NIST11 library used for identification. (C) Chemical structure of the identified compound.

FIGURE S5

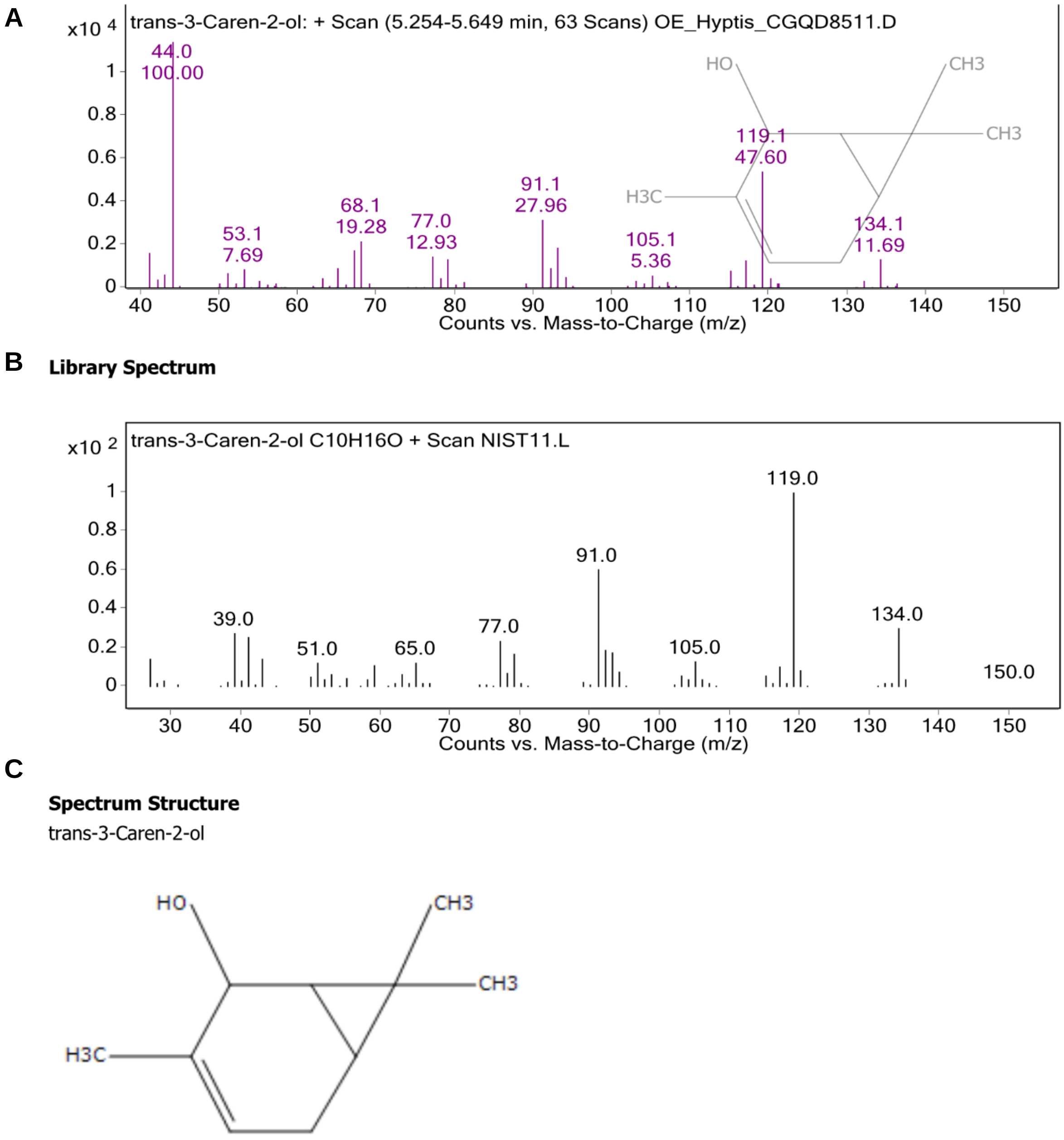

Figure S5 - Mass spectrum of Peak 4 from the TIC chromatogram, assigned to **trans-3-caren-2-ol**. (A) Experimental mass spectrum extracted from the chromatographic analysis. (B) Reference mass spectrum from the NIST11 library used for identification. (C) Chemical structure of the identified compound.

FIGURE S6

A

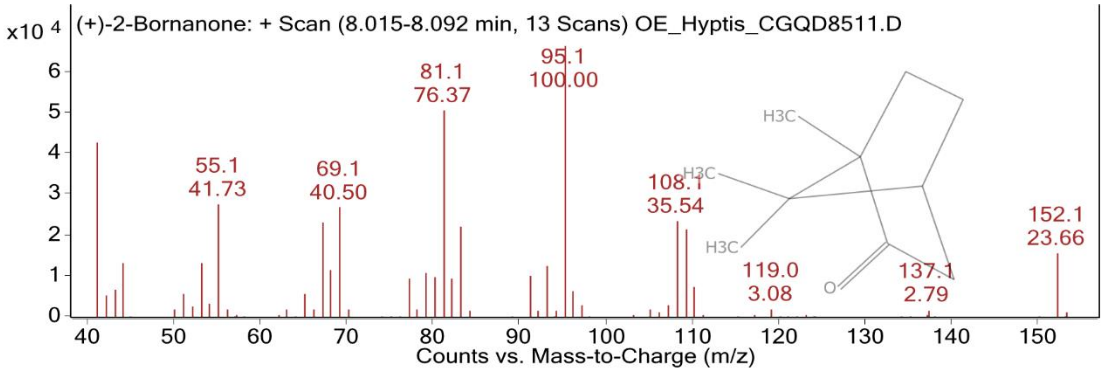

B

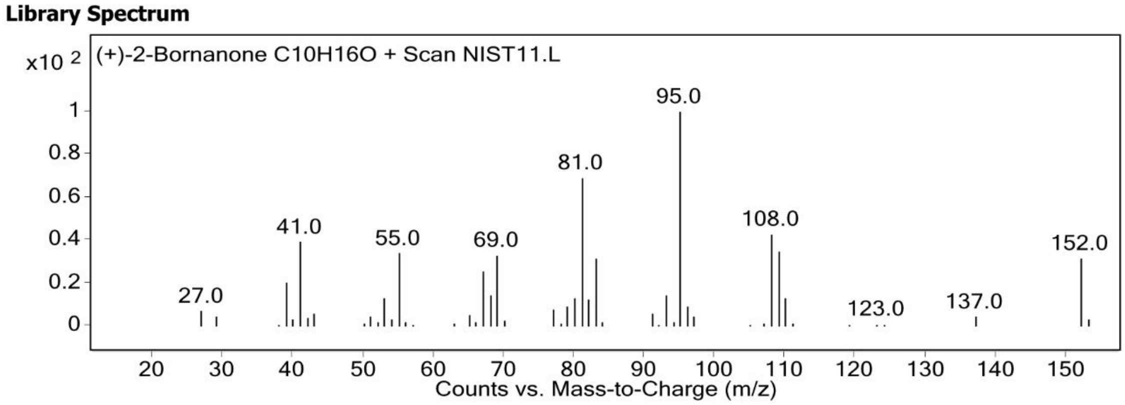

C

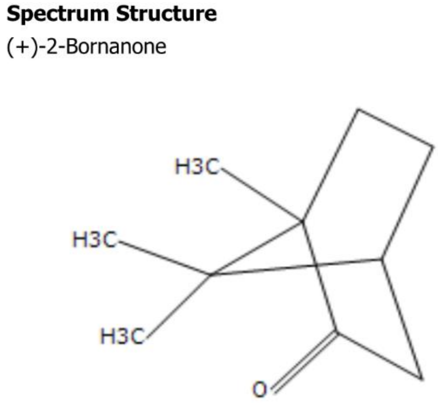

Figure S6 - Mass spectrum of Peak 5 from the TIC chromatogram, assigned to **(+)-2-bornanone**. (A) Experimental mass spectrum extracted from the chromatographic analysis. (B) Reference mass spectrum from the NIST11 library used for identification. (C) Chemical structure of the identified compound.

FIGURE S7

A

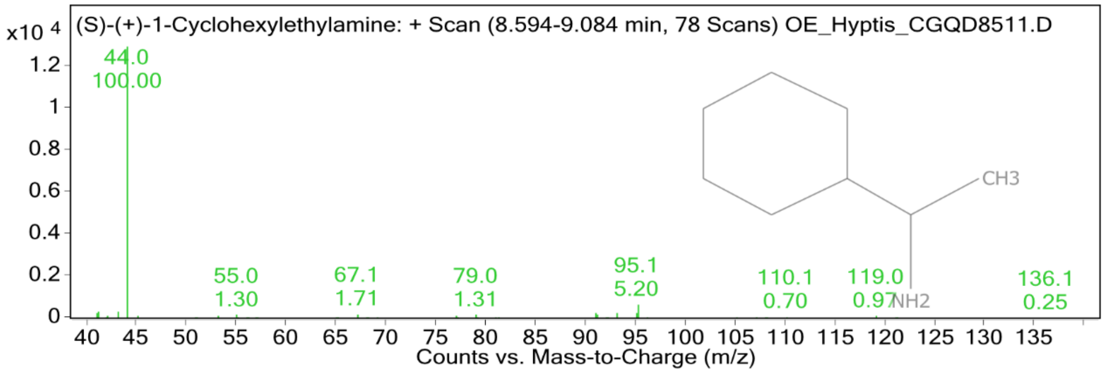

B **Library Spectrum**

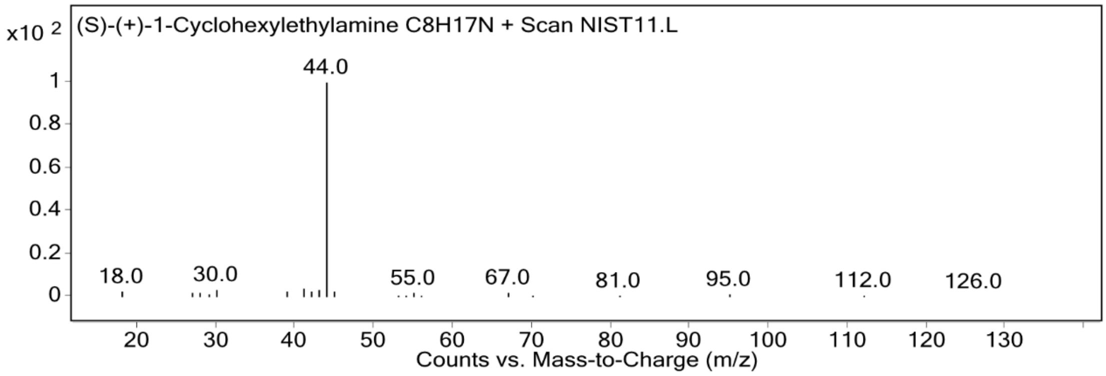

C **Spectrum Structure**

(S)-(+)-1-Cyclohexylethylamine

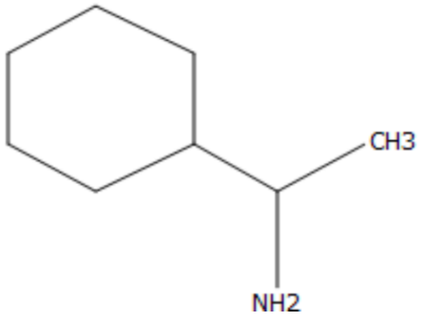

Figure S7 - Mass spectrum of Peak 6 from the TIC chromatogram, assigned to **(S)-(+)1-1-cyclohexylethylamine**. (A) Experimental mass spectrum extracted from the chromatographic analysis. (B) Reference mass spectrum from the NIST11 library used for identification. (C) Chemical structure of the identified compound.

FIGURE S8

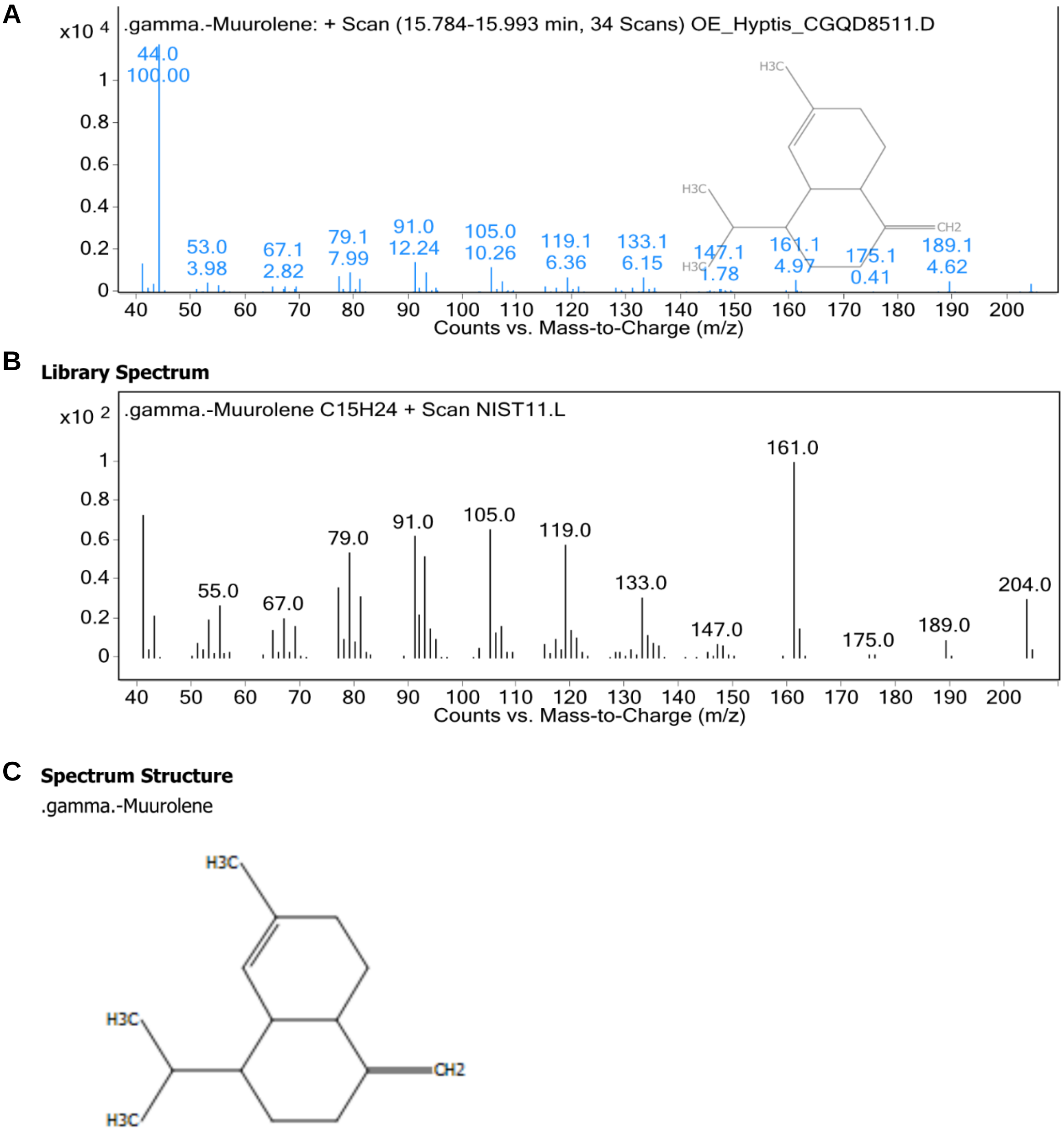

Figure S8 - Mass spectrum of Peak 7 from the TIC chromatogram, assigned **to γ-muurolene**. (A) Experimental mass spectrum extracted from the chromatographic analysis. (B) Reference mass spectrum from the NIST11 library used for identification. (C) Chemical structure of the identified compound.

FIGURE S9

A

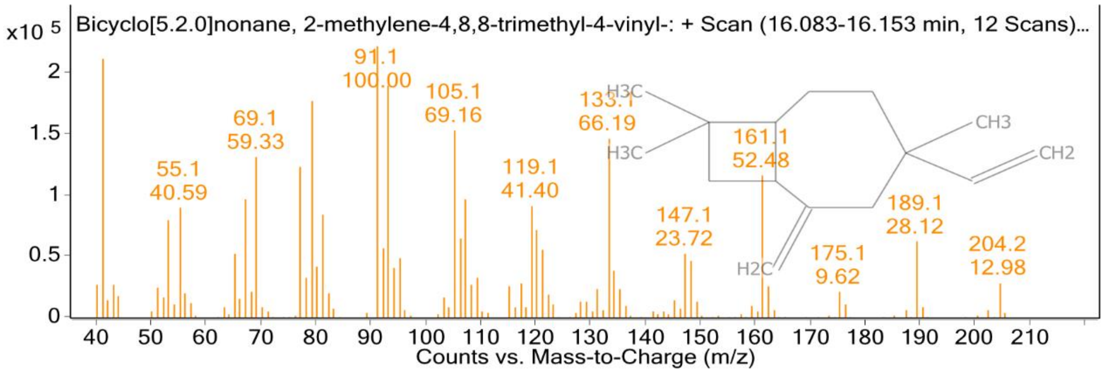

B

Library Spectrum

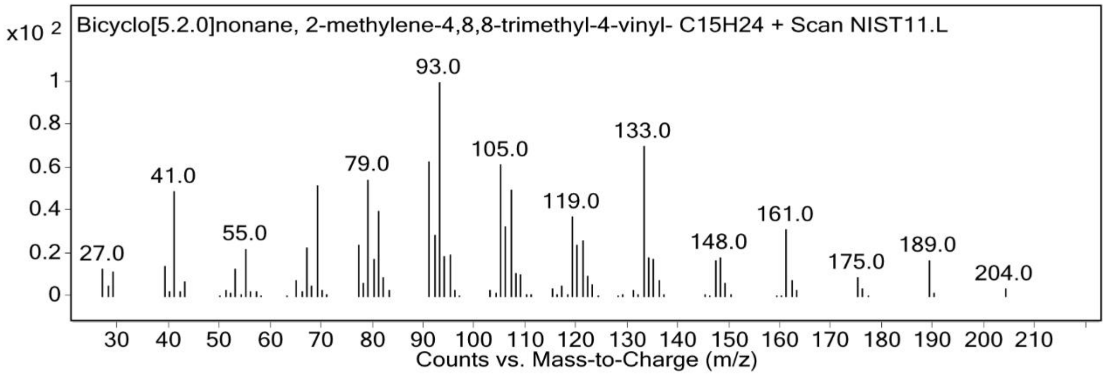

C

Spectrum Structure

Bicyclo[5.2.0]nonane, 2-methylene-4,8,8-trimethyl-4-vinyl-

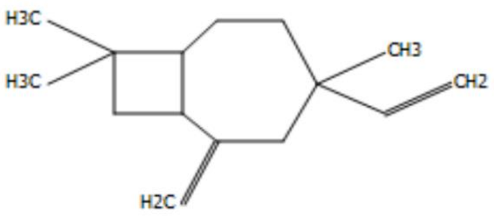

Figure S9 - Mass spectrum of Peak 8 from the TIC chromatogram, assigned to **Bicyclo[5.2.0]nonane, 2-methylene-4,8,8-trimethyl-4-vinyl** (usual synonym: **caryophyllene V1)**. (A) Experimental mass spectrum extracted from the chromatographic analysis. (B) Reference mass spectrum from the NIST11 library used for identification. (C) Chemical structure of the identified compound.

FIGURE S10

A

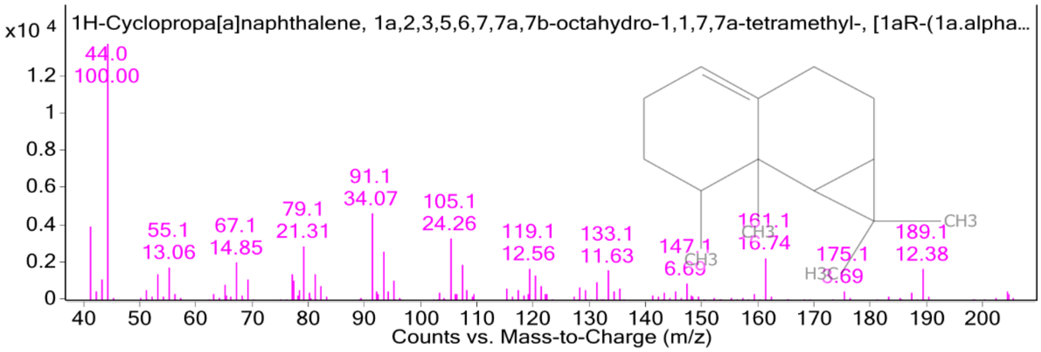

B

Library Spectrum

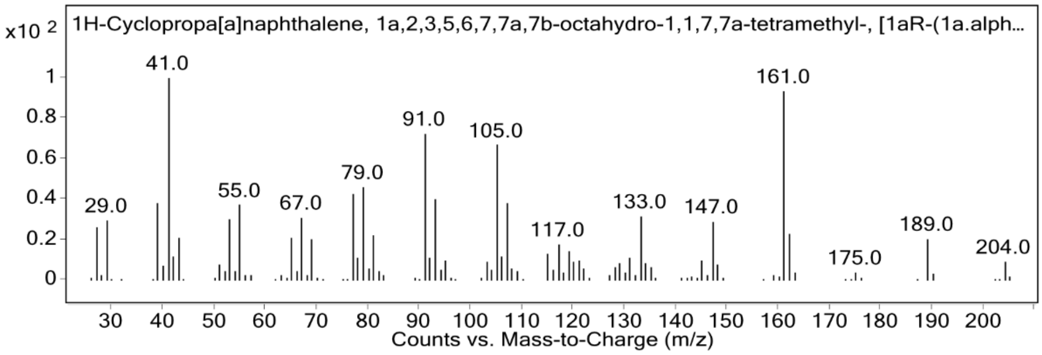

C

Spectrum Structure

1H-Cyclopropa[a]naphthalene, 1a,2,3,5,6,7,7a,7b-octahydro-1,1,7,7a-tetramethyl-, [1aR-(1a.alpha.,7.alpha.,7a.alpha.,7b.alpha.

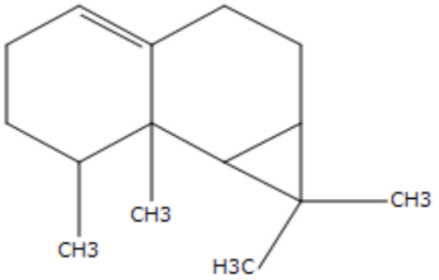

Figure S10 - Mass spectrum of Peak 9 from the TIC chromatogram, assigned to 1H-Cyclopropa[α]naphthalene, 1α,2,3,5,6,7,7α,7β-octahydro-1,1,7,7α-tetramethyl-, [1αR-(1α.α.,7.α.,7α.α.,7β.α.)] (usual synonym: **calarene).** (A) Experimental mass spectrum extracted from the chromatographic analysis. (B) Reference mass spectrum from the NIST11 library used for identification. (C) Chemical structure of the identified compound.

FIGURE S11

A

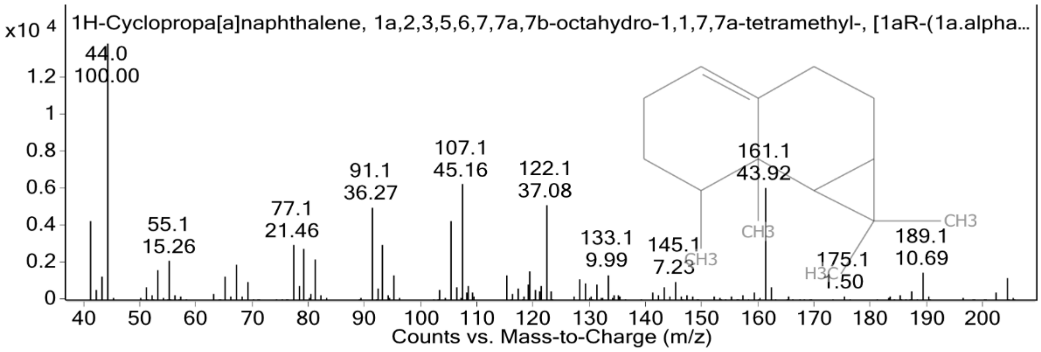

B **Library Spectrum**

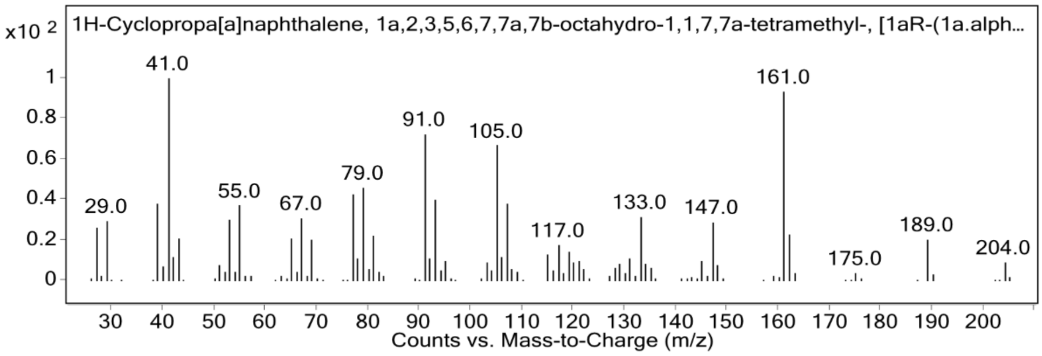

C **Spectrum Structure**

1H-Cyclopropa[a]naphthalene, 1a,2,3,5,6,7,7a,7b-octahydro-1,1,7,7a-tetramethyl-, [1aR-(1a.alpha.,7.alpha.,7a.alpha.,7b.alpha.

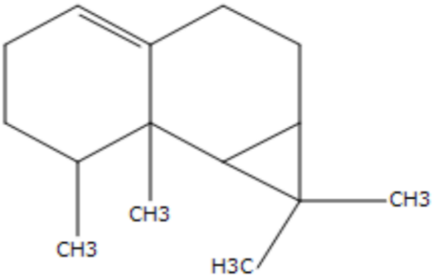

Figure S11 - Mass spectrum of Peak 10 from the TIC chromatogram, assigned to **calarene** for the second time. (A) Experimental mass spectrum extracted from the chromatographic analysis. (B) Reference mass spectrum from the NIST11 library used for identification. (C) Chemical structure of the identified compound.

FIGURE S12

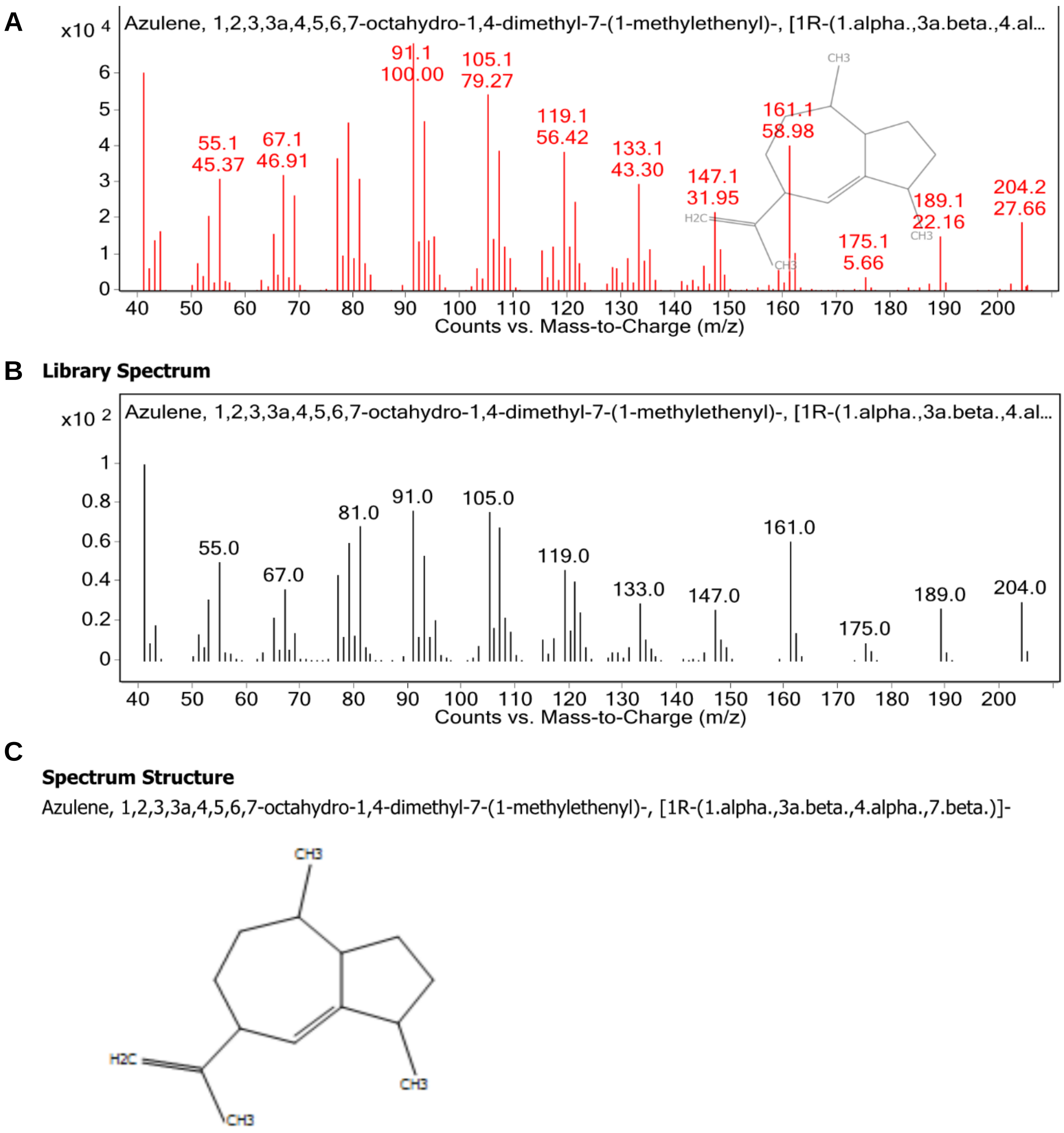

Figure S12 - Mass spectrum of Peak 11 from the TIC chromatogram, assigned to **azulene**. (A) Experimental mass spectrum extracted from the chromatographic analysis. (B) Reference mass spectrum from the NIST11 library used for identification. (C) Chemical structure of the identified compound.

FIGURE S13

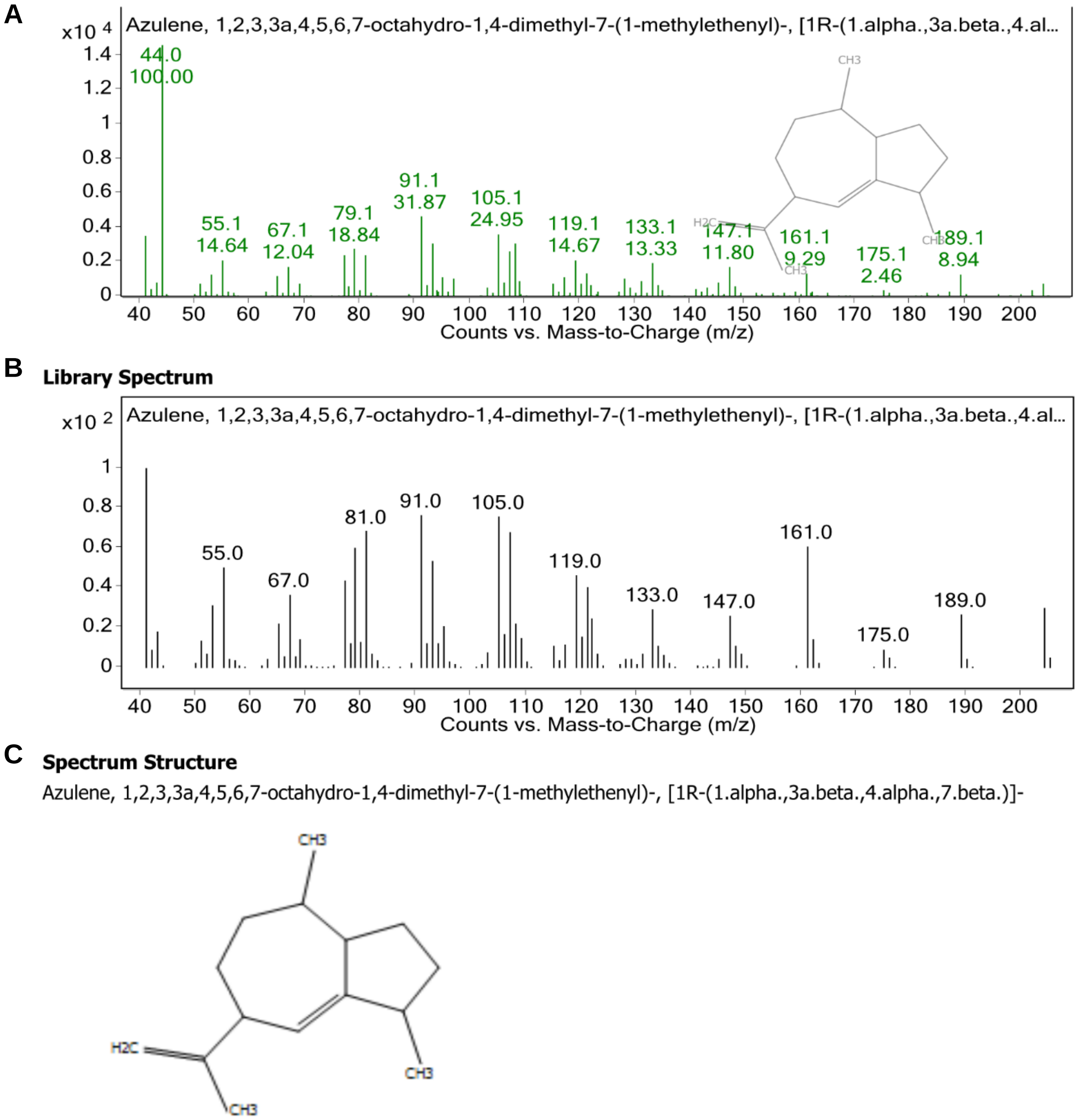

Figure S13 - Mass spectrum of Peak 12 from the TIC chromatogram, assigned to **azulene** for the second time. (A) Experimental mass spectrum extracted from the chromatographic analysis. (B) Reference mass spectrum from the NIST11 library used for identification. (C) Chemical structure of the identified compound.

FIGURE S14

A

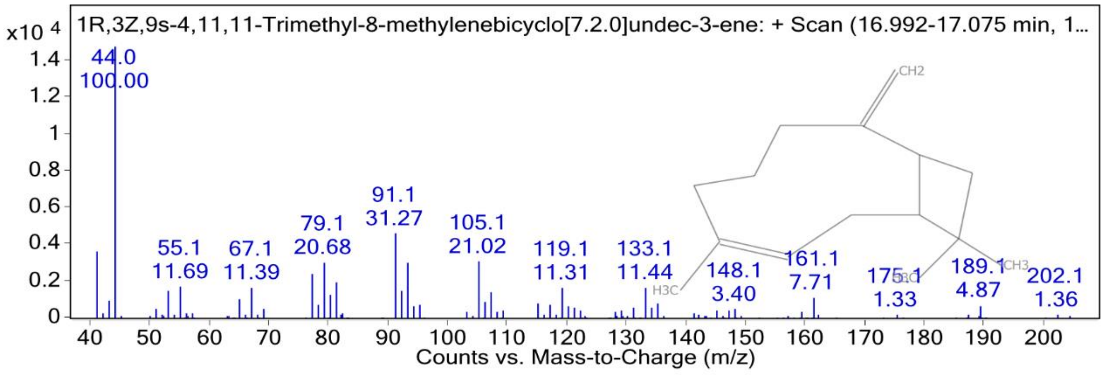

B

Library Spectrum

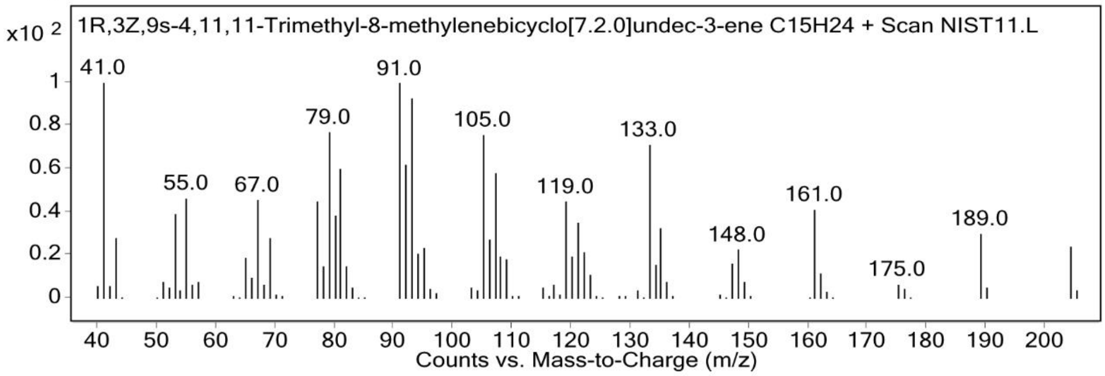

C

Spectrum Structure

1R,3Z,9s-4,11,11-Trimethyl-8-methylenebicyclo[7.2.0]undec-3-ene

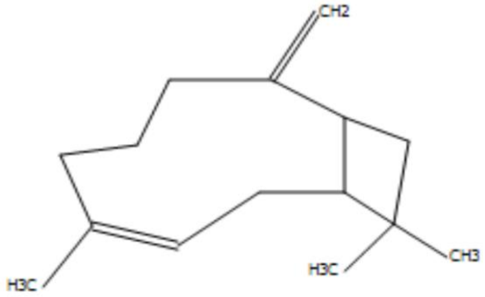

Figure S14 - Mass spectrum of Peak 13 from the TIC chromatogram, assigned to **1R,3Z,9s-4,11,11-Trimethyl-8-methylenebicyclo[7.2.0]undec-3-ene** (usual synonym: **Humulene V1).** (A) Experimental mass spectrum extracted from the chromatographic analysis. (B) Reference mass spectrum from the NIST11 library used for identification. (C) Chemical structure of the identified compound.

FIGURE S15

A

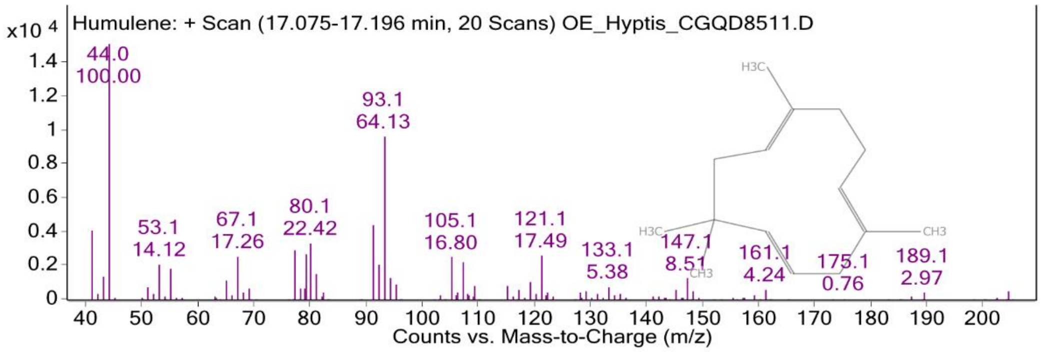

B **Library Spectrum**

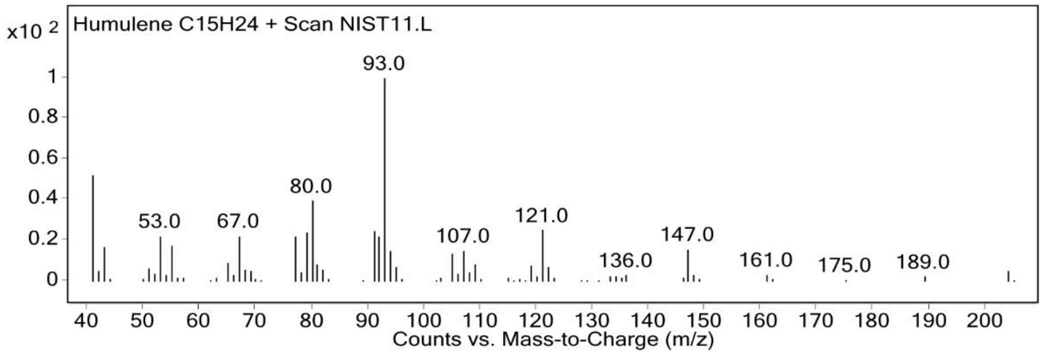

C

Spectrum Structure

Humulene

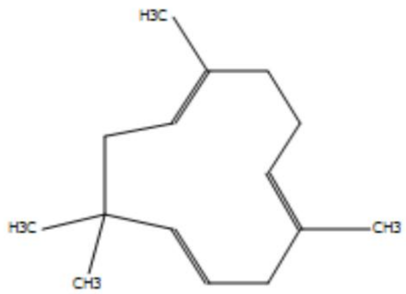

Figure S15 - Mass spectrum of Peak 14 from the TIC chromatogram, assigned to **Humulene**. (A) Experimental mass spectrum extracted from the chromatographic analysis. (B) Reference mass spectrum from the NIST11 library used for identification. (C) Chemical structure of the identified compound.

FIGURE S16

A

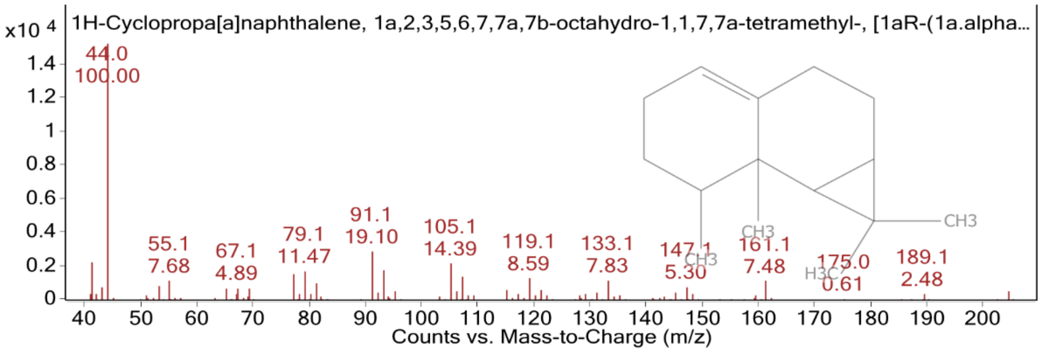

B

Library Spectrum

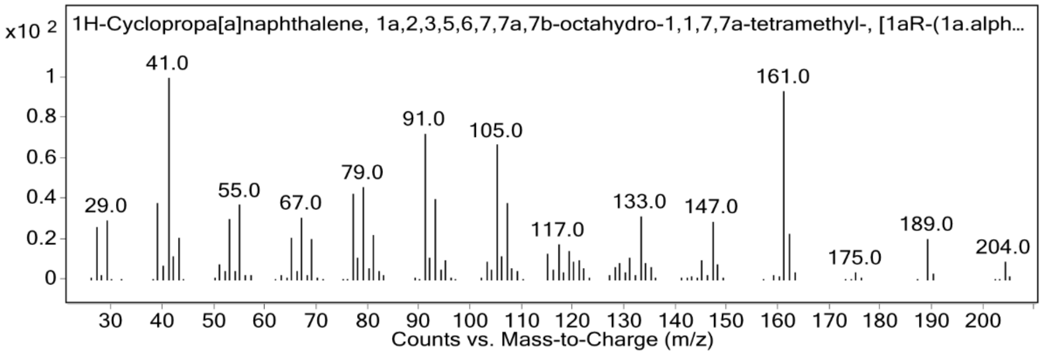

C **Spectrum Structure**

1H-Cyclopropa[a]naphthalene, 1a,2,3,5,6,7,7a,7b-octahydro-1,1,7,7a-tetramethyl-, [1aR-(1a.alpha.,7.alpha.,7a.alpha.,7b.alpha.

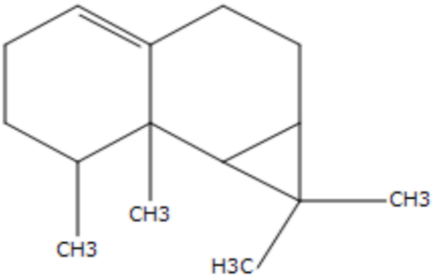

Figure S16 - Mass spectrum of Peak 15 from the TIC chromatogram, assigned to **calarene** for the third time. (A) Experimental mass spectrum extracted from the chromatographic analysis. (B) Reference mass spectrum from the NIST11 library used for identification. (C) Chemical structure of the identified compound.

FIGURE S17

A

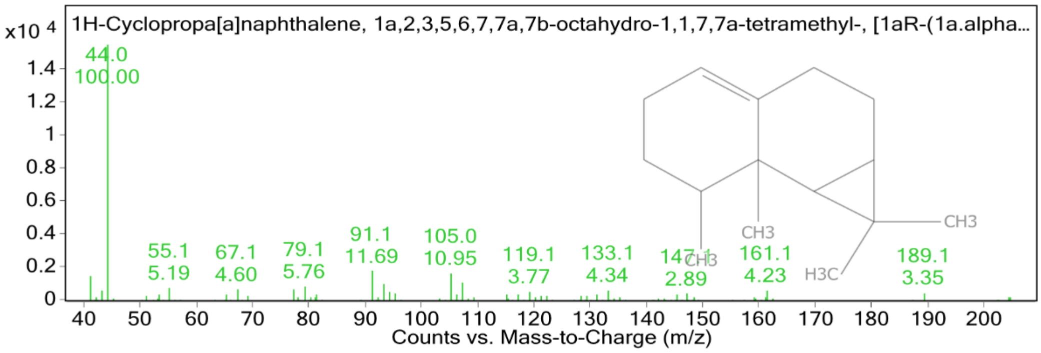

B **Library Spectrum**

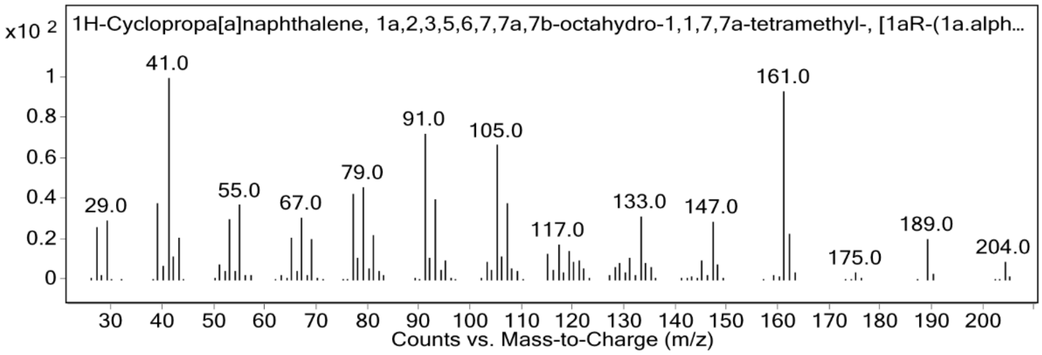

C **Spectrum Structure**

1H-Cyclopropa[a]naphthalene, 1a,2,3,5,6,7,7a,7b-octahydro-1,1,7,7a-tetramethyl-, [1aR-(1a.alpha.,7.alpha.,7a.alpha.,7b.alpha.

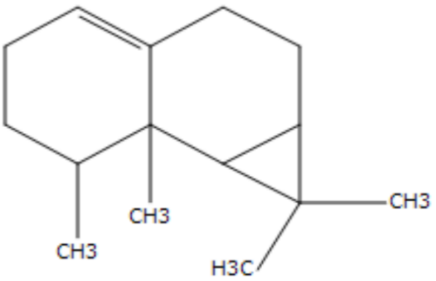

Figure S17 - Mass spectrum of Peak 15 from the TIC chromatogram, assigned to **calarene** for the fourth time. (A) Experimental mass spectrum extracted from the chromatographic analysis. (B) Reference mass spectrum from the NIST11 library used for identification. (C) Chemical structure of the identified compound.

FIGURE S18

A

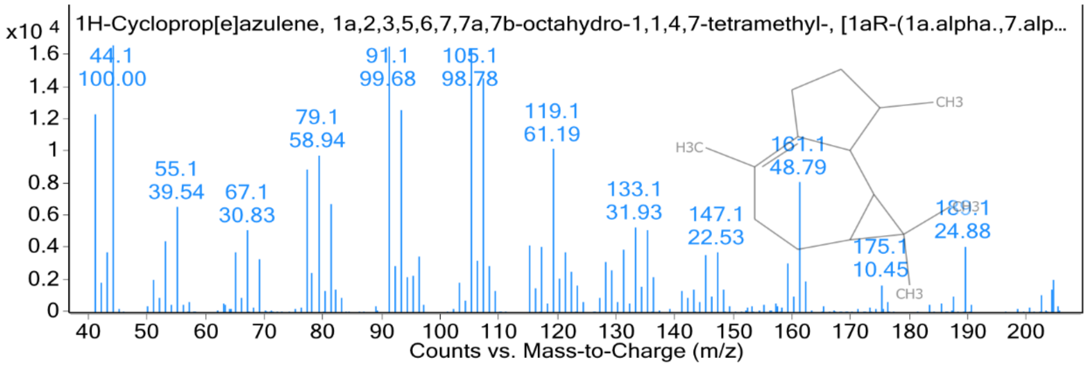

B **Library Spectrum**

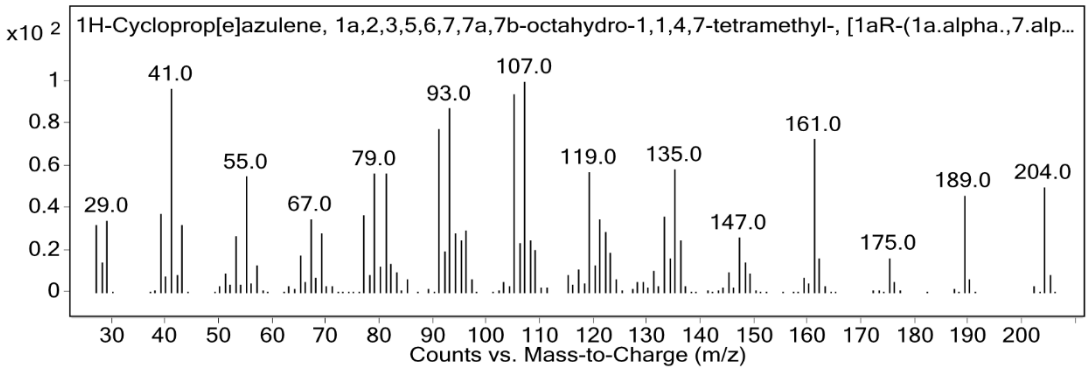

C

Spectrum Structure

1H-Cycloprop[e]azulene, 1a,2,3,5,6,7,7a,7b-octahydro-1,1,4,7-tetramethyl-, [1aR-(1a.alpha.,7.alpha.,7a.beta.,7b.alpha.)]-

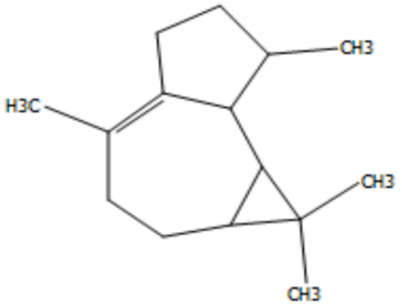

Figure S18 - Mass spectrum of Peak 17 from the TIC chromatogram, assigned to **1H-Cycloprop[e]azulene, 1 α,2,3,5,6,7,7 α,7β-octahydro-1,1,4,7-tetramethyl-, [1 α R-(1α.α.,7.α.,7α.β.,7β.α.)]-** (usual synonym: **(+)-ledene / viridiflorene**). (A) Experimental mass spectrum extracted from the chromatographic analysis. (B) Reference mass spectrum from the NIST11 library used for identification. (C) Chemical structure of the identified compound.

FIGURE S19

A

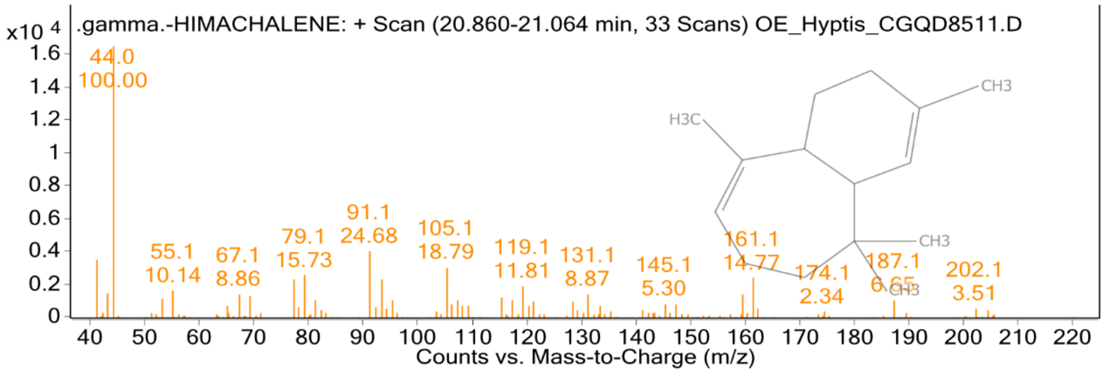

B Library Spectrum

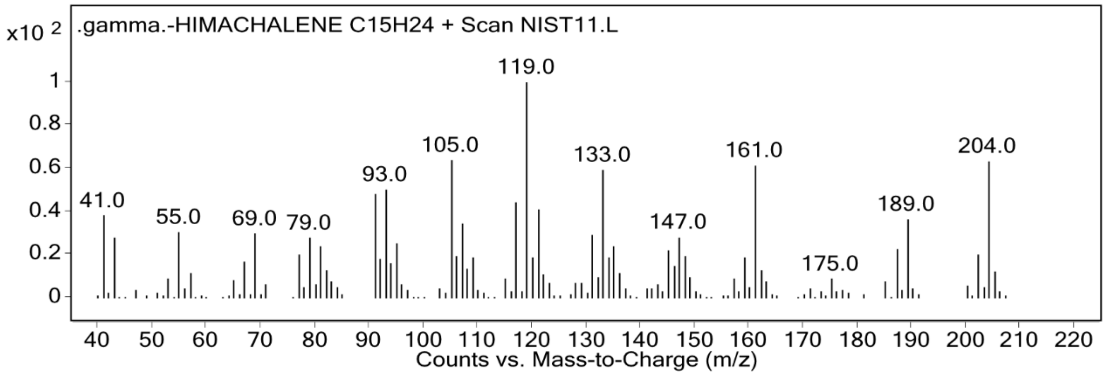

C

Spectrum Structure

.gamma.-HIMACHALENE

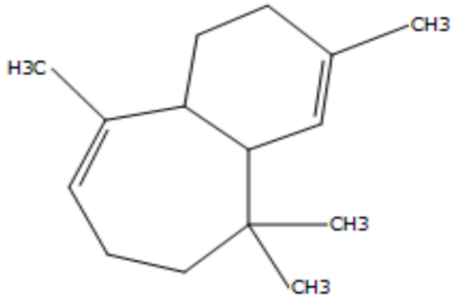

Figure S19 - Mass spectrum of Peak 18 from the TIC chromatogram, assigned to **γ-himachalene.** (A) Experimental mass spectrum extracted from the chromatographic analysis. (B) Reference mass spectrum from the NIST11 library used for identification. (C) Chemical structure of the identified compound.

FIGURE S20

A

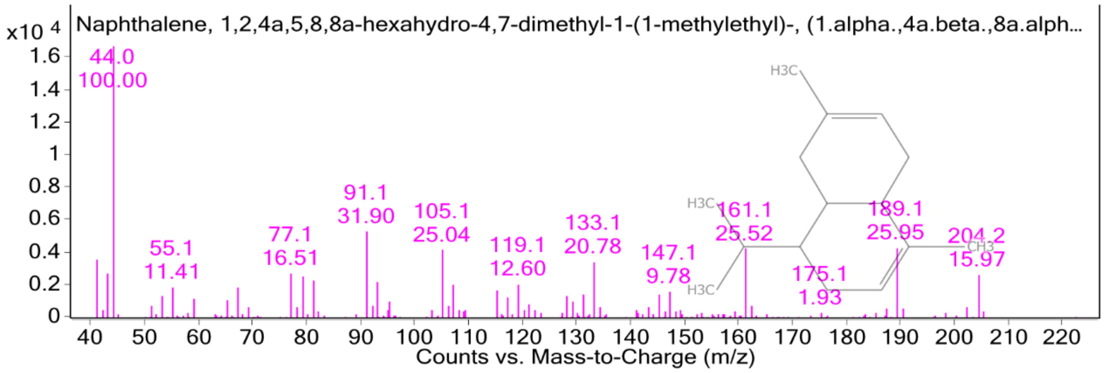

B

Library Spectrum

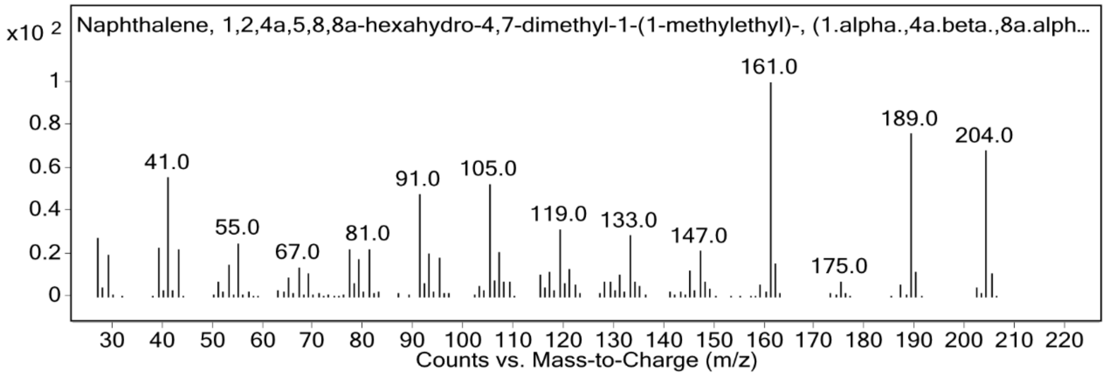

C

Spectrum Structure

Naphthalene, 1,2,4a,5,8,8a-hexahydro-4,7-dimethyl-1-(1-methylethyl)-, (1.alpha.,4a.beta.,8a.alpha.)-(.+/-.)-

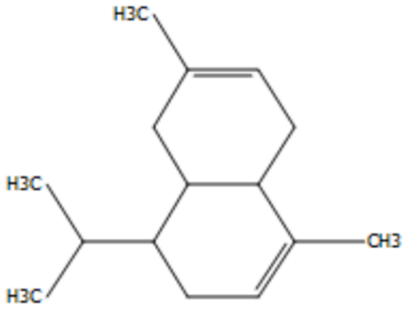

Figure S20 - Mass spectrum of Peak 19 from the TIC chromatogram, assigned to **Naphthalene, 1,2,4α,5,8,8α-hexahydro-4,7-dimethyl-1-(1-methylethyl)-, (1.α.,4α.β.,8α.α.)-(.+/-.)-** (usual synonym: **(±)-cadinene**). (A) Experimental mass spectrum extracted from the chromatographic analysis. (B) Reference mass spectrum from the NIST11 library used for identification. (C) Chemical structure of the identified compound.

FIGURE S21

A

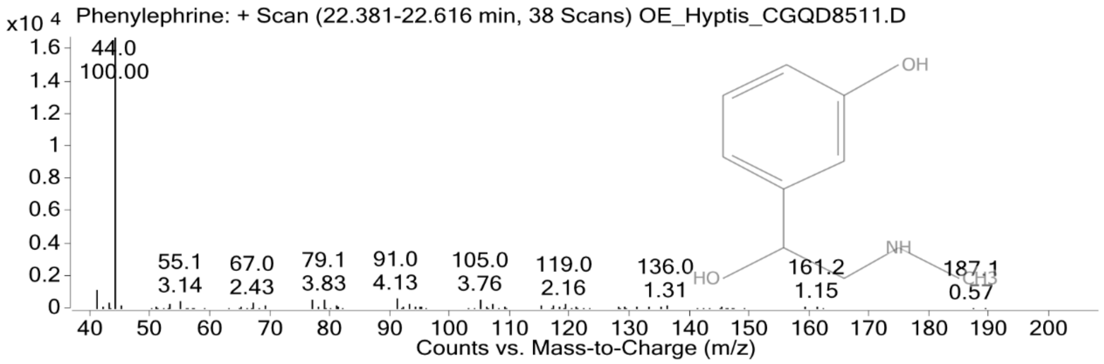

B

Library Spectrum

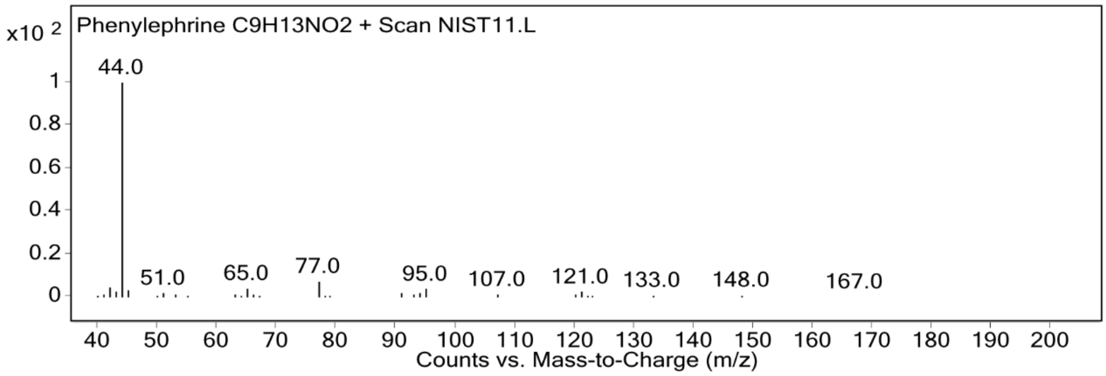

C

Spectrum Structure

Phenylephrine

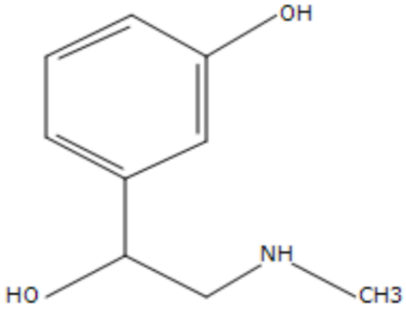

Figure S21 - Mass spectrum of Peak 20 from the TIC chromatogram, assigned to **phenylephrine.** (A) Experimental mass spectrum extracted from the chromatographic analysis. (B) Reference mass spectrum from the NIST11 library used for identification. (C) Chemical structure of the identified compound.

FIGURE S22

A

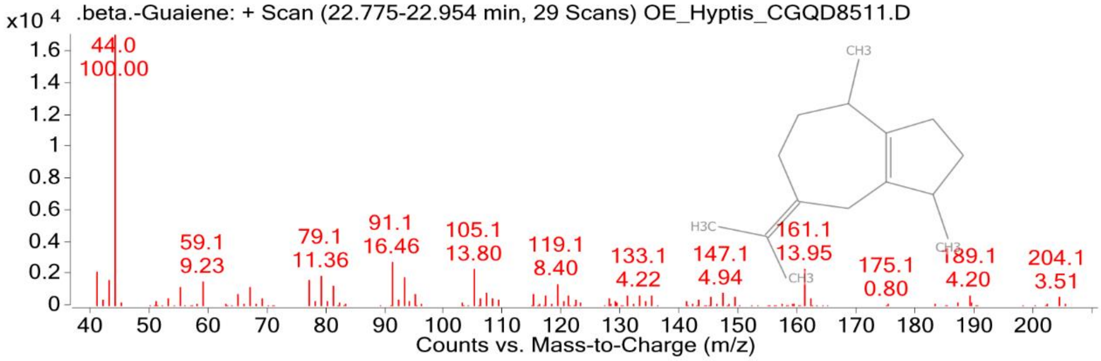

B

Library Spectrum

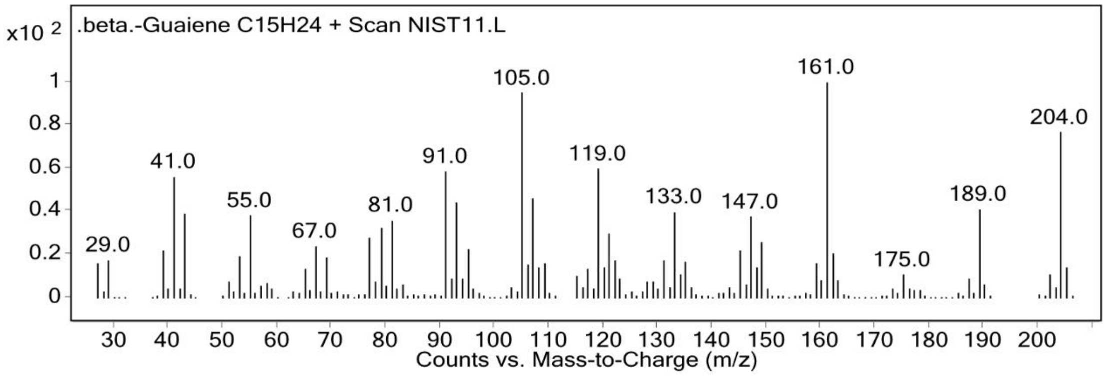

C

Spectrum Structure

.beta.-Guaiene

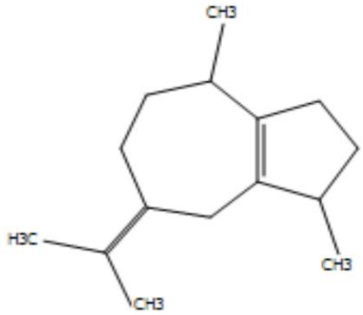

Figure S22 - Mass spectrum of Peak 21 from the TIC chromatogram, assigned to **β-guaiene**. (A) Experimental mass spectrum extracted from the chromatographic analysis. (B) Reference mass spectrum from the NIST11 library used for identification. (C) Chemical structure of the identified compound.

FIGURE S23

A

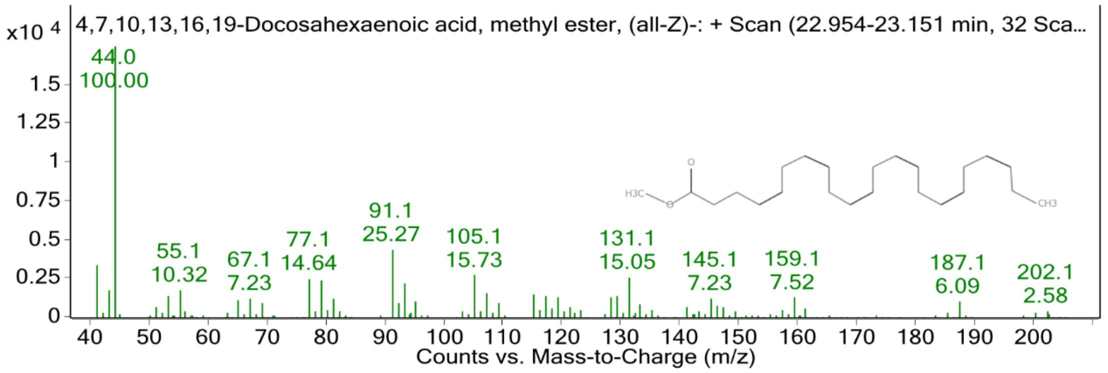

B **Library Spectrum**

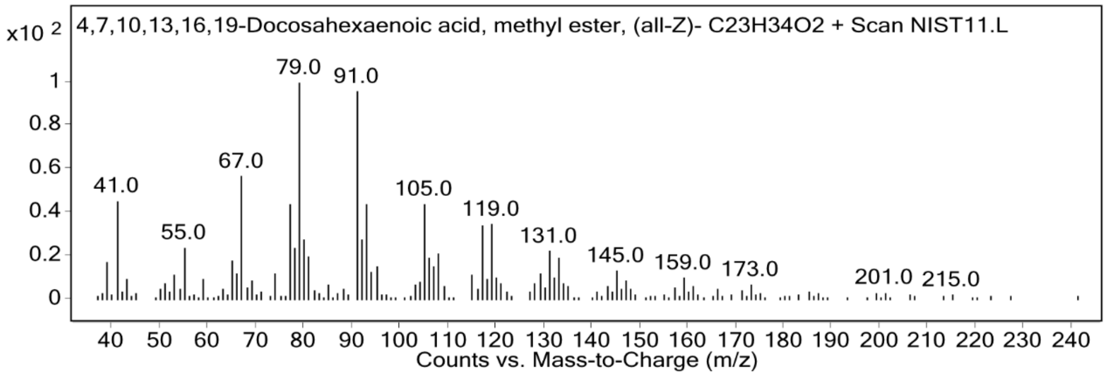

C **Spectrum Structure**

4,7,10,13,16,19-Docosahexaenoic acid, methyl ester, (all-Z)-

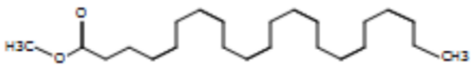

Figure S23 - Mass spectrum of Peak 22 from the TIC chromatogram, assigned to **4,7,10,13,16,19-Docosahexaenoic acid, methyl ester, (all-Z)-**. (A) Experimental mass spectrum extracted from the chromatographic analysis. (B) Reference mass spectrum from the NIST11 library used for identification. (C) Chemical structure of the identified compound.

FIGURE S24

A

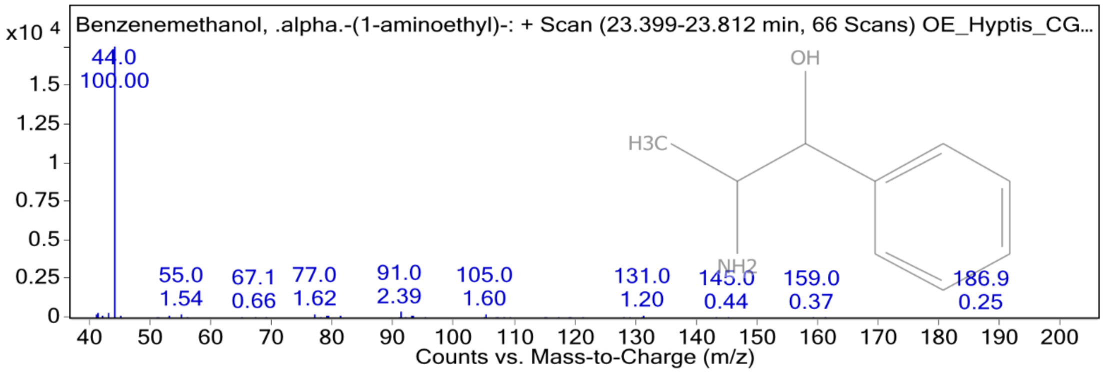

B **Library Spectrum**

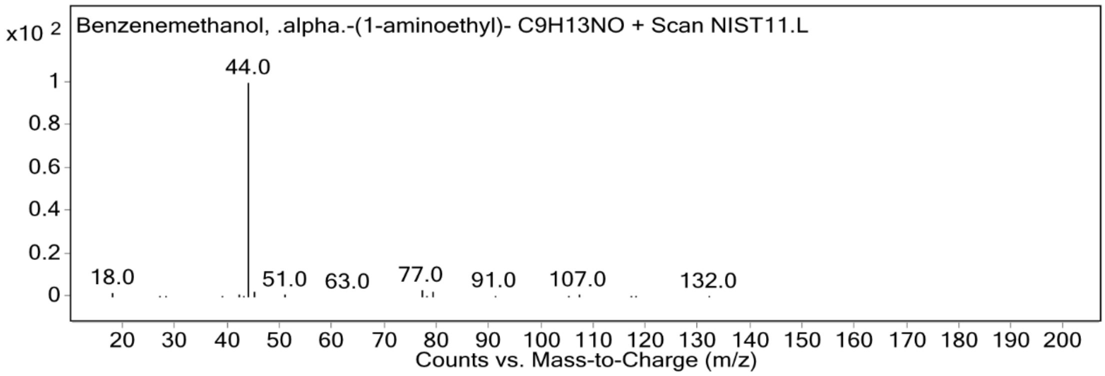

C **Spectrum Structure**

Benzenemethanol, .alpha.-(1-aminoethyl)-

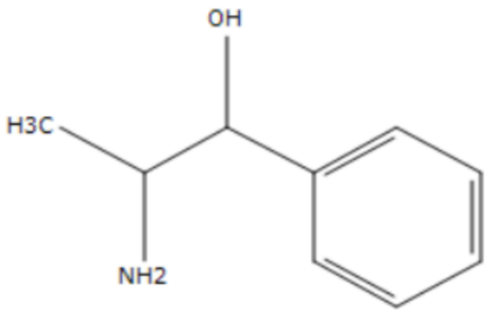

Figure S24 - Mass spectrum of Peak 23 from the TIC chromatogram, assigned to **(1S,2R)-(+)-norephedrine.** (A) Experimental mass spectrum extracted from the chromatographic analysis. (B) Reference mass spectrum from the NIST11 library used for identification. (C) Chemical structure of the identified compound.

TABLE S2

Table S2 Short- and long-range Lennard-Jones interactions, as well as electrostatic forces, were identified in the caryophyllene V1::apo SERT complex. The data were generated using the Arpeggio server (https://biosig.lab.uq.edu.au/arpeggioweb/calculate/). All residues shown in bold correspond to apo SERT, while the numbers indicate the quantity and type of interactions that the caryophyllene V1 ligand maintains with apo SERT.

**Short and long-range Lennard-Jones interactions**																	
**Types of interactions**		**apo SERT**	**Tyr^{95}**	**Ala^{169}**	**Ile^{172}**	**Tyr^{176}**	**Val^{343}**	**Ser^{336}**	**Leu^{337}**	**Gly^{338}**	**Phe^{341}**	**Ser^{438}**	**Ser^{439}**	**Gly^{442}**	**Ala^{173}**	**Leu^{443}**	**Total interactions**
**Clashes de Van der Waals**	Undefined-vdwclash								1								**1**
**Proximal**	Undefined-proximal	**Caryophyllene V1**	13	6	**7**	1	2	3	6	5	7	12	11	11	3	2	**89**
	Weakpolar-proximal												1				**1**
	Hydrophobic-proximal		1	2	4	1	3				3						**14**

**Electrostaic force**																	
**Types of interactions**		**apo SERT**	**Tyr^{95}**	**Ala^{169}**	**Ile^{172}**	**Tyr^{176}**	**Val^{343}**	**Ser^{336}**	**Leu^{337}**	**Gly^{338}**	**Phe^{341}**	**Ser^{438}**	**Ser^{439}**	**Gly^{442}**	**Ala^{173}**	**Leu^{443}**	**Total interactions**
**Polar contact**	Weakpolar-VdW	**Caryophyllene V1**											1				**1**
**Carbonyl**	Carbonyl-proximal										1						**1**
